# Dietary Modulation of Gut Microbiota and Metabolome Shapes Growth Performance in *Thamnaconus septentrionalis*

**DOI:** 10.3390/ani16091312

**Published:** 2026-04-24

**Authors:** Qinmei Fang, Ling Ke, Li Bian, Shuigen Li, Hongshu Chi, Yongcong Chen, Ximin Qiu, Shaohua Shi, Siqing Chen

**Affiliations:** 1 Biotechnology Research Institute, Fujian Academy of Agricultural Sciences, Fuzhou 350011, China; 2 State Key Laboratory of Mariculture Biobreeding and Sustainable Goods, Yellow Sea Fisheries Research Institute, Chinese Academy of Fishery Sciences, Qingdao 266071, China; 3 Fujian Fisheries Technology Extension Center, Fuzhou 350011, China

**Keywords:** intestinal microbiome, feed conversion, 16S rRNA sequencing, marine carnivorous fish, machine learning prediction

## Abstract

*Thamnaconus septentrionalis* is an economically important marine aquaculture species in China. However, the acceptance rate of formulated feeds in commercial farming is only 30–40%, substantially lower than the 80–90% achieved with fresh feeds, which severely constrains the intensive development of this industry. The gut microbiota-mediated regulatory mechanisms underlying the effects of different feed types on growth performance remain unclear, limiting the precise development of efficient formulated feeds. This study established four feed types (commercial pellet feed K, custom-formulated feed P, frozen shrimp X, and fresh fish meat Y) through a 60-day feeding trial. Growth performance data, 16S rRNA sequencing, and untargeted metabolomics were analyzed. Random Forest-Partial Least Squares Regression models were employed to identify key microbial-metabolite features. Results indicated that the Y group exhibited the optimal feed conversion ratio (1.14), with intestinal Firmicutes abundance (45.3%) significantly higher than the K group (28.5%). Short-chain fatty acid levels increased by more than 350-fold, enriching short-chain fatty acid-producing bacteria such as *Lactobacillus* and *Faecalibacterium*. The P group, formulated with high fishmeal content (40%), achieved performance levels comparable to the Y group across most indicators. Machine learning models identified key microbial-metabolite features predicting growth performance, providing a multi-omics framework for developing efficient formulated feeds for marine carnivorous fish.

## Simple Summary

Many farmed marine fish prefer fresh food over manufactured feed, which limits sustainable aquaculture. This study investigated why different feeds affect growth differently in an economically important fish species, the greenfin horse-faced filefish. Scientists compared four feed types: commercial pellets, a specially formulated high-fishmeal pellet, frozen shrimp, and fresh fish meat. After 60 days, fish fed fresh fish meat grew best and converted feed into body mass most efficiently. Their gut bacteria were healthier and produced more beneficial short-chain fatty acids. The specially formulated feed performed almost as well as fresh fish meat, far better than the commercial pellets. Using machine learning, the team identified specific bacteria and gut metabolites linked to faster growth. These findings provide a scientific basis for developing better manufactured feeds, reducing dependence on fresh fish. This will help marine aquaculture become more efficient and sustainable, lowering costs and environmental impact while supporting global food production.

## 1. Introduction

The rapid expansion of global aquaculture has imposed severe challenges on sustainable feed resources. As wild fishery resources approach their ecological carrying capacity, aquaculture has become the dominant pathway to meet global seafood demand, accounting for over 50% of global seafood supply [1]. However, formulated feeds heavily depend on fishmeal, driving up costs to 50–70% of total expenditure [2,3]. For marine carnivorous fish, this challenge is amplified: many emerging species exhibit poor acceptance of formulated feeds and remain dependent on fresh or frozen feeds, impeding the standardization of aquaculture practices [4,5].

Among these emerging marine carnivorous species facing dietary adaptation challenges, *Thamnaconus septentrionalis* stands out as a representative case. With annual aquaculture production exceeding 15,000 tons along China’s eastern coast [6], this species faces a critical challenge in feed development: the acceptance rate of formulated feeds is only 30–40%, substantially lower than the 80–90% achieved with fresh feeds [6]. This results in a 20–30 day extension in culture period and a 40–50% increase in feed costs [7], severely constraining the transition from traditional extensive to modern intensive aquaculture systems. More critically, nutritional research on *T. septentrionalis* remains scarce, and the underlying mechanisms by which different feed types influence growth performance remain unclear, severely limiting the precise development of efficient formulated feeds.

Traditional fish nutrition research has primarily focused on the direct effects of feed nutrient composition on growth performance [8,9]. Marine-sourced proteins, such as fishmeal, significantly outperform plant proteins due to their amino acid profiles being highly matched to fish requirements [10], while the supply level of n-3 long-chain polyunsaturated fatty acids (EPA and DHA) directly determines growth rate and feed conversion efficiency in marine fish [11]. However, such traditional nutrition studies generally regard the fish intestine as a passive nutrient absorption organ, neglecting the active regulatory role of the intestine as a complex ecosystem in nutritional metabolism. With advances in high-throughput sequencing, the gut microbiome has emerged as a key regulator of host growth and health through nutrient degradation, metabolite synthesis, and immune modulation [12,13], providing a new perspective for understanding how different feed types regulate growth performance.

Feed composition is one of the primary drivers shaping fish gut microbial community structure [14]. Different nutritional substrates can selectively enrich or inhibit specific microbial populations, thereby influencing host physiological functions. For instance, replacing fishmeal with plant proteins in Atlantic salmon (*Salmo salar*) diets leads to increased Proteobacteria abundance and decreased Firmicutes, consequently triggering intestinal inflammation and growth suppression [15]. Similarly, dietary carbohydrate levels in channel catfish (*Ictalurus punctatus*) alter carbon metabolism functional genes in gut microbiota, modulating host energy utilization [16]. However, current research predominantly remains at the level of correlations between microbial community shifts and growth phenotypes, and the scientific question of how feed types regulate host growth performance through gut microbiota-mediated metabolism remains unresolved.

Metabolomics provides a complementary approach to microbiome analysis by capturing the functional output of microbial communities. Gut microbiota-derived metabolites, including short-chain fatty acids, amino acid derivatives, and bile acids, serve as signaling molecules that directly modulate host physiology [17,18]. In aquaculture species, metabolomic studies have begun to reveal how dietary changes alter the gut metabolic landscape: fishmeal replacement with plant proteins disrupts bile acid and amino acid metabolism in rainbow trout (*Oncorhynchus mykiss*) [19], and soybean meal-induced enteritis in Atlantic salmon (*Salmo salar*) is associated with altered taurine and inositol levels [20]. However, these metabolomic studies predominantly rely on pairwise correlations, which cannot distinguish causal from coincidental associations or identify the key microbial-metabolite combinations that drive growth outcomes [13]. Moreover, research on marine carnivorous fish remains extremely limited, and how their gut microbiota and metabolome respond to different feed types is poorly understood.

Integrating multi-omics data with machine learning approaches offers a new avenue to overcome these limitations. Machine learning algorithms such as Random Forest can identify key features contributing to growth performance from hundreds of microbial taxa and metabolites through feature importance scoring [21,22,23], yet the application of such integrative approaches in aquaculture remains in its infancy. To date, no study has systematically integrated 16S rRNA sequencing, metabolomics, and machine learning to elucidate the mechanisms by which feeds modulate fish growth performance through gut microbiota.

Based on this background, the present study employed juvenile *T. septentrionalis* as the research model and designed four representative feed types: commercial pelleted formulated feed (K group), custom-formulated feed (P group, 40% fishmeal + 15% soybean meal), frozen shrimp (X group), and fresh fish meat (Y group), conducting a 60-day feeding trial. The study aimed to (1) evaluate the effects of four feed types on growth performance, muscle nutritional quality, and gut microbial community structure of *T. septentrionalis*; (2) identify key metabolites and enriched pathways associated with dietary treatments; and (3) develop multi-omics machine learning models to identify core microbial-metabolite features predicting growth outcomes, thereby providing a scientific basis for developing efficient formulated feeds for marine carnivorous fish.

## 2. Materials and Methods

### 2.1. Ethics Statement

All animal experimental procedures were approved by the Animal Ethics Committee of FAAS (approval No.: BI-AEC-20230308, Date: 26 October 2023) and strictly followed the Guidelines for Ethical Review of Laboratory Animal Welfare (GB/T 35892-2018) [24]. Procurement of *T. septentrionalis* required no special permits, as this species is not listed on endangered species registers, and collection did not occur in protected areas. Anesthesia employed a graded MS-222 (tricaine methanesulfonate, Sigma-Aldrich, St. Louis, MO, USA) protocol to ensure animal welfare. Experimental facilities (Greenfin Pufferfish Aquaculture Research Platform, License No.: 202301) were certified for laboratory animal use.

### 2.2. Experimental Animals and Acclimation

Juvenile *T. septentrionalis* were purchased from Zhaoan Marine Aquaculture Farm, Zhangzhou City, Fujian Province (23.71 °N, 117.17 °E) and acclimated for 14 days in an indoor recirculating aquaculture system (total volume 5 m^3^, equipped with mechanical filtration, biofiltration, and UV sterilization modules). During acclimation, fish were fed commercial pelleted feed (crude protein ≥ 45%, crude lipid ≥ 10%) twice daily (08:00 and 17:00) at 3–5% body weight. A total of 1200 juvenile *T. septentrionalis* were initially procured to accommodate transportation mortality, acclimation stress, and rigorous health screening. During the 14-day acclimation period, fish exhibiting abnormal swimming behavior, external lesions, or poor feeding response were examined for common bacterial (*Vibrio*, *Aeromonas*) and parasitic pathogens by gill and skin smears under light microscopy; affected individuals were removed from the trial. Following acclimation, 300 healthy juveniles with uniform size (initial body weight 25.3 ± 1.2 g, body length 10.2 ± 0.5 cm) were systematically selected through random stratified sampling for the formal feeding trial, ensuring statistical power and biological replication across all treatment groups.

### 2.3. Experimental Design and Culture Management

This study employed a completely randomized single-factor design with four feed treatments (Table S1): (1) K group (pelleted formulated feed), commercial marine fish pellets (brand: Bluefish Pellets; manufacturer: Fujian Dabeinong Huayou Aquatic Technology Group Co., Ltd., Zhao’an, China); a detailed composition is in Table S1; (2) P group (custom-formulated feed), formulated according to *T. septentrionalis* nutritional requirements (with high fishmeal content (40%) and fish oil (5%) to provide high-quality protein and sufficient n-3 long-chain polyunsaturated fatty acids) with high fishmeal content (40%) and fish oil (5%) to provide high-quality protein and sufficient n-3 long-chain polyunsaturated fatty acids. Detailed ingredient compositions of all feed groups are provided in Table S1. Both K and P groups were manufactured as extruded pellets using identical processing parameters (detailed in Supplementary Table S1); (3) X group (shrimp), frozen Chinese shrimp slices (purchased from Zhaoan Seafood Wholesale Market) representing natural crustacean feeds; (4) Y group (fresh fish), fresh small yellow croaker chunks representing natural fish feeds.

Proximate composition of the four feeds was analyzed following [25] standard methods [25]. The proximate composition of all four feeds is presented in Table S2. Given the substantial differences in moisture content among the four feed types (K: 10.5%, P: 10.8%, X: 76.2%, Y: 78.5%), all feed conversion ratio (FCR) calculations were standardized on a dry-matter basis: FCR_DM = dry matter feed intake/wet weight gain, to enable valid inter-group comparisons. Groups X and Y were purchased twice weekly, stored at −20 °C, thawed to room temperature before feeding, and cut into 1–2 cm palatable pieces. Each treatment included three replicate tanks (12 tanks total), with 25 juveniles randomly assigned per tank (initial density 2108 g/m^3^). Culture tanks were cylindrical polyethylene vessels (diameter 80 cm, height 60 cm, effective volume 300 L). The system employed flow-through aeration with an inflow rate of 2.0 L/min and a daily water exchange of approximately 30% (90 L/tank). Culture conditions were strictly maintained: water temperature 22 ± 1 °C, salinity 28–32‰, dissolved oxygen ≥ 6.0 mg/L, pH 7.8–8.2, photoperiod 12L:12D (light intensity 300–500 lux). Fish were fed to satiation twice daily at 08:00 and 17:00, with actual feed intake of 2.5–4.0% body weight. Uneaten feed was removed 1 h post-feeding and recorded. The same feeding protocol (fed to apparent satiation twice daily) was maintained consistently throughout the entire 60-day trial for all treatment groups, with feed amounts adjusted based on observed consumption to ensure ad libitum feeding.

### 2.4. Sample Collection Strategy

Samples were collected on Days 1 (baseline), 30 (mid-term), and 60 (endpoint) following 24 h fasting. Stratified sampling ensured statistical power and adequate representation: Day 1 sampling included 3 fish per group (12 fish total) to characterize initial population; Day 30 intestinal sampling included 3 fish per replicate tank × 3 replicates = 9 fish/group (36 fish total); Day 60 intestinal sampling included 4 fish per replicate tank × 3 replicates = 12 fish/group (48 fish total). Fish were individually immersed in an MS-222 bath solution (tricaine methanesulfonate, 150 mg/L, buffered with sodium bicarbonate to neutral pH) until cessation of opercular movement (approximately 2–3 min), confirming deep anesthesia. Fish were then euthanized by rapid cervical dislocation while still under anesthesia, following AVMA Guidelines for the Euthanasia of Animals (2020). After measuring body weight and morphometric parameters, the abdominal cavity was dissected to isolate intestinal tissue, which was gently rinsed with ice-cold sterile PBS (pH 7.4). The intestine was opened longitudinally, and intestinal contents (approximately 0.2–0.5 g per fish) were scraped into 2 mL cryotubes using pre-sterilized spatulas, immediately flash-frozen in liquid nitrogen, and stored at −80 °C. Simultaneously, 5 g of dorsal white muscle was excised, skin and red muscle removed, rinsed with PBS, blotted dry, flash-frozen, and stored at −80 °C for nutritional analysis. All operations were conducted on ice under sterile conditions, with the interval from euthanasia to sample freezing controlled within 10 min.

### 2.5. Growth Performance and Morphometric Measurements

Growth performance was measured on Days 1, 30, and 60 [26]. After 24 h fasting, fish were lightly anesthetized with MS-222 (100 mg/L), individually weighed using an electronic balance, and measured for total length, body length, body height, body thickness, head length, and eye diameter using digital calipers. From the measured fish, a subset was sacrificed for organ weight determination (8 fish/tank on Day 30, 10 fish/tank on Day 60; of these, 3 and 4 fish/tank, respectively, were used for intestinal sample collection as described in Section 2.4). Weight gain rate (WGR, %) was calculated as (final weight − initial weight)/initial weight × 100. Specific growth rate (SGR, %/day) was calculated as (ln final weight − ln initial weight)/days × 100. Feed conversion ratio (FCR) was total feed intake/total weight gain. Survival rate (%) was (final fish number/initial fish number) × 100. Morphometric indices included hepatosomatic index (HSI, liver weight/body weight × 100), viscerosomatic index (VSI, viscera weight/body weight × 100), and condition factor (CF, g/cm^3^, body weight/body length^3^ × 100).

### 2.6. Muscle Nutritional Composition Analysis

Samples were ground into powder in liquid nitrogen. For amino acid analysis, 1.0 g of sample was extracted with 5% trichloroacetic acid, and seventeen free amino acids were quantified by an automated amino acid analyzer with ninhydrin derivatization. For fatty acid analysis, 2.0 g of muscle was extracted with chloroform-methanol (2:1, *v*/*v*), methylated, and analyzed by gas chromatography (GC-2010 Plus, Shimadzu, Kyoto, Japan; SP-2560 column, Agilent Technologies, Santa Clara, CA, USA). Results were expressed as a percentage of total fatty acids [27].

### 2.7. Intestinal Microbiota 16S rRNA Gene Sequencing

Total DNA from intestinal contents (*n* = 3 per group at Day 30 and *n* = 4 per group at Day 60; total 84 samples across all groups and time points) was extracted using FastDNA SPIN Kit for Soil (MP Biomedicals, Irvine, CA, USA), which employs a bead-beating lysis method effective for diverse sample matrices including intestinal contents (widely adopted in fish gut microbiome studies), and verified by 1% agarose gel electrophoresis. The V3-V4 hypervariable region of bacterial 16S rRNA gene was amplified using primers 338F (5′-ACTCCTACGGGAGGCAGCAG-3′) and 806R (5′-GGACTACHVGGGTWTCTAAT-3′) [28]. PCR employed 2 × Phusion High-Fidelity PCR Master Mix (Thermo Fisher Scientific, Waltham, MA, USA) with the following cycling conditions: 98 °C for 3 min; 25 cycles of 98—10 s, 55—30 s, 72—45 s; and final extension at 72 °C for 5 min. PCR products were pooled in equimolar ratios, and libraries were constructed using TruSeq DNA PCR-Free Sample Preparation Kit (Illumina, San Diego, CA, USA) and sequenced on Illumina NovaSeq 6000 platform (PE250) with target depth ≥ 50,000 reads/sample.

Raw sequencing data underwent quality filtering with fastp (v0.23.2) and paired-end read merging with FLASH (v1.2.11). The QIIME2 platform (2023.5) with the DADA2 algorithm performed sequence denoising, chimera removal, and ASV clustering. Taxonomic annotation was conducted against the SILVA database (Release 138) (confidence threshold 0.7). α-diversity indices (Chao1, Shannon, Simpson) were calculated using the diversity plugin at 95% of the minimum depth. β-diversity was visualized by principal coordinate analysis (PCoA) based on Bray–Curtis distance matrices. Between-group differences were tested by permutational multivariate analysis of variance (PERMANOVA). PICRUSt2 predicted functional gene composition and mapped to the KEGG database [29].

### 2.8. Untargeted Metabolomics Analysis of Intestinal Contents

Fifty milligrams of intestinal contents were mixed with 400 μL pre-chilled methanol-water (4:1, *v*/*v*) containing 0.02 mg/mL internal standard (L-2-chlorophenylalanine). Two stainless steel beads (5 mm diameter) were added, and samples were homogenized at 60 Hz for 2 min. After ultrasonic extraction (300 W, 5 s on/5 s off cycles) at 4 °C for 30 min, samples were incubated at −20 °C for 30 min to precipitate proteins. Following centrifugation (12,000 rpm, 10 min, 4 °C), supernatants were transferred to vials. To minimize temporal variation, all intestinal content samples within each time point were collected within a single day and processed simultaneously. Quality control (QC) samples were prepared by pooling 20 μL from each sample and injecting every 10 samples to assess system stability and reproducibility. Analysis employed ultra-high performance liquid chromatography-quadrupole time-of-flight mass spectrometry (UPLC-Q-TOF-MS) following established protocols [30]. LC conditions: ACQUITY UPLC BEH C18 column (100 mm × 2.1 mm, 1.7 μm), column temperature 40 °C, injection volume 5 μL, mobile phases A (0.1% formic acid in water) and B (acetonitrile), flow rate 0.4 mL/min, with gradient elution optimized for polar metabolite separation. MS conditions: electrospray ionization in positive and negative modes, capillary voltage 3.0 kV (positive)/−2.5 kV (negative), source temperature 120 °C, desolvation temperature 450 °C, mass range *m*/*z* 50–1200.

Raw data were processed by Progenesis QI for peak extraction, alignment, normalization, and metabolite identification. Primary identification used accurate mass with retention time matching to authentic standards; secondary identification used accurate mass with MS/MS fragmentation patterns matched to databases (HMDB, METLIN, KEGG). Orthogonal partial least squares discriminant analysis (OPLS-DA) [31] was performed with 7-fold cross-validation to assess model quality (R^2^Y and Q^2^ parameters), with 200 permutation tests validating against overfitting. Differential metabolites were selected by VIP > 1.0, |log_2_FC| ≥ 0.5, and FDR < 0.05. KEGG pathway enrichment analysis employed hypergeometric distribution testing with Benjamini–Hochberg FDR correction (FDR < 0.05 considered significant) [32].

### 2.9. Multi-Omics Integration and Machine Learning Analysis

Correlations among gut microbiota, metabolite levels, and growth performance were evaluated by Spearman rank correlation based on individual sample data (Day 30: *n* = 36; Day 60: *n* = 48) rather than group means. Input matrices included core microbial abundance, core metabolite concentrations, and growth performance metrics (WGR, SGR, FCR). Mantel test evaluated overall associations between microbial community structure and metabolite composition, with microbial distance matrix calculated by the Bray–Curtis distance and metabolite distance matrix by Euclidean distance (data log_2_-transformed and Pareto-scaled), quantifying association strength by Pearson correlation with permutation testing for significance.

To identify core microbial-metabolite features influencing growth performance, an exploratory machine learning-based feature selection and regression framework was constructed [33]. Original features included 35 core genera and metabolites. Engineered features captured nonlinear and synergistic effects, including microbial ratio features, microbe–metabolite interaction terms, and quadratic terms, totaling 54 features. All features were standardized by RobustScaler to mitigate outlier effects. Random Forest algorithm (1000 trees) ranked features by mean impurity decrease, selecting core features (top 40%). To validate selection stability, feature selection was repeated through 100 bootstrap iterations, retaining only features selected in ≥70% of iterations. Partial least squares regression (PLSR) models were built using selected features as predictors and growth metrics as responses, with 5-fold cross-validation repeated 5 times to assess generalization. The component number was optimized by grid search. To address the limited sample size, bootstrap resampling (1000 iterations, which constituted the data augmentation strategy referenced in the limitations) was employed for internal validation, and a hold-out validation set (20% of samples, stratified by treatment group) was reserved prior to model training to provide an independent assessment of model generalizability. Model performance was evaluated by goodness-of-fit (R^2^), root mean squared error (RMSE), and mean absolute error (MAE), with permutation tests validating significance. For comparison, additional models (ElasticNet, XGBoost, Support Vector Regression) were also evaluated. All analyses were performed in Python (v3.9) with scikit-learn (v1.2.0) for Random Forest feature selection and PLSR modeling.

### 2.10. Statistical Analysis

Statistical analyses were performed using SPSS 26.0 (IBM, Armonk, NY, USA). For growth performance and morphometric analyses, the tank was treated as the true experimental unit (*n* = 3 tanks per treatment); individual fish measurements within each tank were averaged to obtain tank means before statistical analysis. Data are presented as mean ± SEM. Following the Shapiro–Wilk normality test and Levene’s homogeneity of variance test, two-way ANOVA evaluated main effects of feed type and sampling time and their interaction for tank-level growth data. For individual-level data (muscle nutritional composition), linear mixed-effects models (LMMs) were employed with feed type and sampling time as fixed effects and tank as a random effect to account for the hierarchical data structure, with Tukey’s HSD test for multiple comparisons. All statistical tests involving multiple comparisons employed Benjamini–Hochberg FDR correction, with FDR < 0.05 as the significance threshold. For single comparisons without multiple testing (e.g., two-way ANOVA main effects, Mantel tests), *p* < 0.05 indicated significance. Note that because fish were destructively sampled at each time point (different individuals at Days 1, 30, and 60), traditional repeated-measures designs on the same individuals were not applicable. Instead, time was modeled as a fixed factor in both the ANOVA and LMM frameworks described above, with tank serving as the unit linking observations across the hierarchical structure. Alpha diversity indices were compared among groups using the Kruskal–Wallis test followed by Dunn’s post hoc test with FDR correction. Beta diversity differences were assessed by PERMANOVA (999 permutations) based on the Bray–Curtis distance.

## 3. Results and Discussion

### 3.1. Effects of Feed Types on Growth Performance and Morphology of T. septentrionalis

#### 3.1.1. Temporal Dynamics of Growth Indices

**Figure 1:**
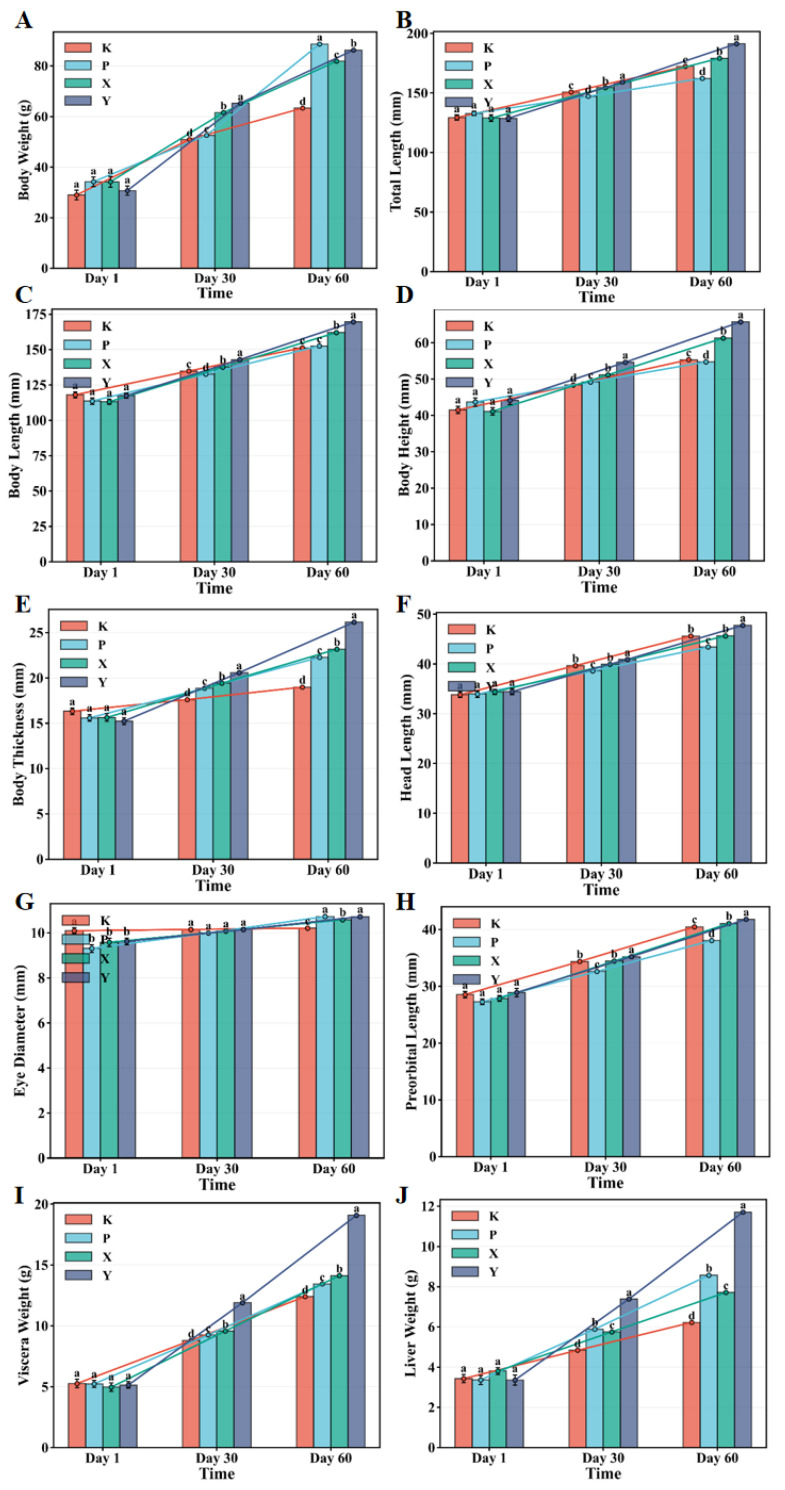
Temporal dynamics of growth and morphometric parameters in *T. septentrionalis* fed different feed types. (**A**) Body weight, (**B**) total length, (**C**) body length, (**D**) body height, (**E**) body thickness, (**F**) head length, (**G**) eye diameter, (**H**) pre-orbital head length, (**I**) viscera weight, (**J**) liver weight. K: commercial pelleted feed; P: custom-formulated feed; X: frozen shrimp; Y: fresh fish. Data are presented as mean ± SEM (*n* = 30). Different letters indicate significant differences among groups at the same timepoint (Tukey’s HSD, *p* < 0.05). Statistical comparisons for feed effects were performed using tank means (*n* = 3 per treatment) as the experimental unit; individual data points are shown for visualization of biological variability.

No significant differences in initial body weight (K: 25.2 g, P: 25.4 g, X: 25.3 g, Y: 25.5 g, *p* = 0.89), total length (K: 10.1 cm, P: 10.2 cm, X: 10.3 cm, Y: 10.2 cm, *p* = 0.92), or condition factor (K: 2.42, P: 2.45, X: 2.43, Y: 2.46, *p* = 0.87) were observed among the four treatment groups on Day 1 (Table S3), ensuring proper experimental design and eliminating potential confounding from initial size heterogeneity. Body weight increased significantly in all groups over time, with feed type-dependent growth differences becoming progressively evident (Figure 1A). By Day 30, the Y group exhibited significantly higher body weight than the K group (*p* < 0.01). By Day 60, inter-group differences further expanded, with the Y group achieving the highest final body weight (71.5 ± 2.3 g, weight gain 46.0 g) followed by P (68.7 ± 2.1 g, weight gain 43.3 g), both significantly higher than the K group (58.3 ± 1.8 g, weight gain 33.1 g; *p* < 0.001), while the X group (65.2 ± 2.0 g, weight gain 39.9 g) remained intermediate. Two-way ANOVA revealed a highly significant interaction effect between feed type and culture time on body weight (F = 18.53). Total length and body length followed patterns consistent with body weight (Figure 1B,C). At Day 60, the Y group displayed significantly greater total length than the K and P groups (*p* < 0.01), while both X and Y groups exceeded the K group in body length (*p* < 0.01). Body height at Day 60 was significantly greater in the Y group (64.80 ± 0.90 mm, height gain 20.70 mm) and X group (62.63 ± 1.11 mm, height gain 21.51 mm) than in the K group (54.57 ± 0.75 mm, height gain 13.06 mm) and P group (55.46 ± 0.86 mm, height gain 11.77 mm) (*p* < 0.01; Figure 1D,E, Table S3). Body thickness showed similar patterns. Notably, cranial features (head length, pre-orbital head length, eye diameter) showed relatively minor inter-group variation (Figure 1F–H), with eye diameter remaining stable throughout the culture period and exhibiting no inter-group differences, suggesting minimal influence from feed type. Visceral organ weights increased significantly with culture time, with the Y group showing significantly higher total viscera weight and liver weight than other groups at Day 60 (*p* < 0.001) (Figure 1I,J).

#### 3.1.2. Growth Performance Parameter Evaluation

**Figure 2:**
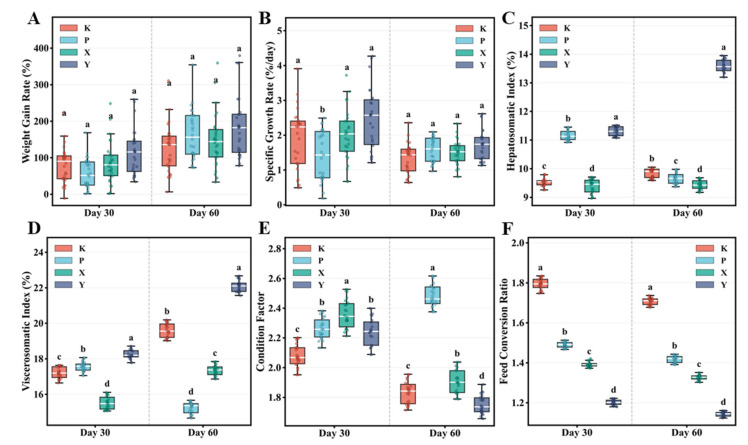
Effects of different feed types on growth performance parameters in *T. septentrionalis*. (**A**) Weight gain rate, (**B**) specific growth rate, (**C**) hepatosomatic index, (**D**) viscerosomatic index, (**E**) condition factor, (**F**) feed conversion ratio. Box plots show interquartile ranges, white center lines represent medians, whiskers indicate extreme values, and dots represent individual data points (*n* = 30). Different letters indicate significant differences among groups at the same timepoint (*p* < 0.05). Group abbreviations are as in Figure 1. Feed conversion ratio was calculated on a dry-matter basis. Statistical analyses used tank means (*n* = 3 per treatment) as experimental units.

WGR and SGR showed no significant inter-group differences (Figure 2A,B, Table S4), though the Y group exhibited numerically higher values at Day 60. FCR (calculated on a dry-matter basis to account for differences in feed moisture content; see Methods) exhibited significant inter-group differences at all time points (Figure 2F). At both Day 30 and Day 60, the Y group showed significantly lower FCR than the other three groups (*p* < 0.001), with the K group displaying the highest values and all four groups differing significantly. Lower FCR values indicate superior feed conversion efficiency, with the Y group achieving optimal feed utilization at both time points. At Day 60, final body weights were K group 58.3 g, P group 68.7 g, X group 65.2 g, and Y group 71.5 g, corresponding to WGR of 131.2%, 170.5%, 157.7%, and 180.4%, respectively. Survival rates remained high across all groups (K: 96.0%, P: 97.3%, X: 94.7%, Y: 98.7%), with no significant inter-group differences (*p* = 0.32), indicating that all feed types supported fish welfare throughout the 60-day trial. Hepatosomatic index (HSI), reflecting liver development and nutritional reserves (Figure 2C), was significantly higher in the Y and P groups than in the K and X groups at Day 30 (*p* < 0.01), while only the Y group maintained significantly elevated HSI at Day 60 (*p* < 0.001). Viscerosomatic index (VSI) displayed differential patterns across time points (Figure 2D): at Day 30, the Y, P, and K groups significantly exceeded the X group (*p* < 0.01), while at Day 60, the Y and K groups were significantly higher than the X and P groups (*p* < 0.001). Condition factor (CF) showed relatively minor inter-group variation (Figure 2E), with the P group significantly exceeding other groups at Day 60 (*p* < 0.05).

### 3.2. Effects of Feed Types on Muscle Nutritional Quality

#### 3.2.1. Muscle Proximate Composition

**Figure 3:**
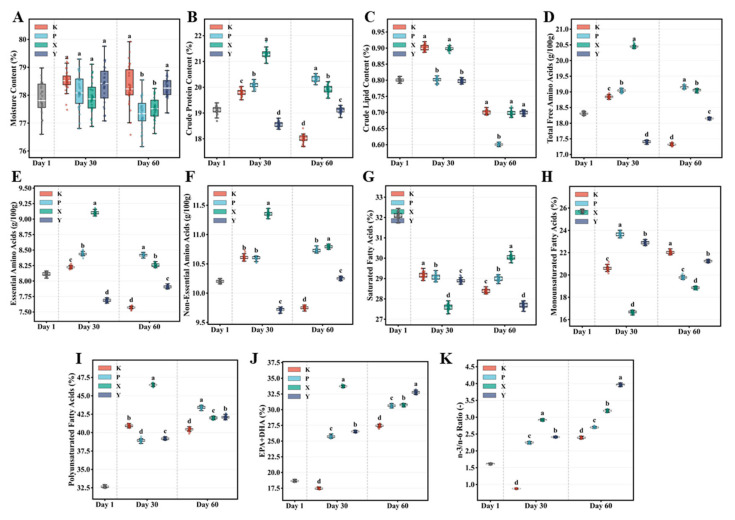
Effects of different feed types on muscle nutritional quality in *T. septentrionalis*. (**A**–**C**) Muscle moisture, crude protein, and crude lipid content; (**D**–**F**) total free amino acids, essential amino acids, and non-essential amino acids content; (**G**–**I**) saturated fatty acids, monounsaturated fatty acids, and polyunsaturated fatty acids content; (**J**,**K**) EPA + DHA content and n-3/n-6 ratio. All data represent estimated marginal means from linear mixed-effects models (LMM) with feed type and time as fixed effects and tank as a random effect. Different letters indicate significant differences among groups at the same timepoint (Tukey-adjusted, *p* < 0.05). White center lines in box plots represent medians. Group abbreviations are as in Figure 1.

Muscle moisture content remained relatively stable across the experimental period (Figure 3A, Table S5). Crude protein content (Figure 3B) was highest in the X group at Day 30, significantly exceeding other groups (*p* < 0.01); at Day 60, the P and X groups significantly surpassed the Y and K groups (*p* < 0.01). Notably, crude protein content in the K group declined significantly from Day 1 to Day 60 (*p* < 0.01). Muscle crude lipid content remained low across all groups, consistent with the characteristics of *T. septentrionalis* as a low-fat carnivorous fish (Figure 3C).

#### 3.2.2. Muscle Free Amino Acid Composition

Total free amino acid (TFAA) content analysis revealed that feed type significantly influenced muscle amino acid accumulation (Figure 3D; Table S6). At Day 30, the X group exhibited significantly higher TFAA than other groups (*p* < 0.01), while at Day 60, the P and X groups significantly exceeded the Y and K groups (*p* < 0.05). TFAA content in both K and Y groups declined below initial levels by Day 60, indicating these feeds failed to effectively maintain muscle free amino acids. Essential amino acid (EAA) content followed similar trends (Figure 3E): at Day 60, the P group showed the highest EAA content, significantly exceeding the K and Y groups (*p* < 0.01). Non-essential amino acids (NEAAs) at Day 60 were significantly higher in the X and P groups than in the K group (*p* < 0.01) (Figure 3F). Detailed analysis of 17 individual amino acids (Table S6) revealed significant inter-group differences in umami amino acids (Asp, Glu) and sweet amino acids (Gly, Ala, Ser), with Glu content in the P and X groups significantly exceeding the K group at Day 60 (*p* < 0.01).

#### 3.2.3. Muscle Fatty Acid Composition

As shown in Figure 3G–K and Table S7, saturated fatty acids (SFAs), monounsaturated fatty acids (MUFAs), and polyunsaturated fatty acids (PUFAs) showed distinct distribution patterns among groups. From Day 1 to Day 60, PUFA content increased significantly in all groups, indicating continuous accumulation of muscle n-3 and n-6 PUFAs over culture time (Figure 3I). Functional n-3 long-chain polyunsaturated fatty acid (EPA + DHA) content was significantly influenced by feed type (Figure 3J). At Day 30, the X group displayed significantly higher EPA + DHA than other groups (*p* < 0.01), while at Day 60, the Y group achieved the highest levels, significantly exceeding the K and P groups (*p* < 0.05). Importantly, the X and Y groups (fresh feed groups) maintained significantly higher EPA + DHA levels than the K group throughout the culture period. At Day 60, the Y group exhibited the highest n-3/n-6 ratio, significantly exceeding the K and P groups (*p* < 0.01) (Figure 3K). The n-3/n-6 ratio in the K group increased from Day 30 to Day 60, indicating muscle fatty acid composition shifted toward healthier n-3 enrichment, though absolute levels remained significantly lower than those of the fresh feed groups.

### 3.3. Effects of Feed Types on Gut Microbial Community Structure

#### 3.3.1. Gut Microbiota Diversity

**Figure 4:**
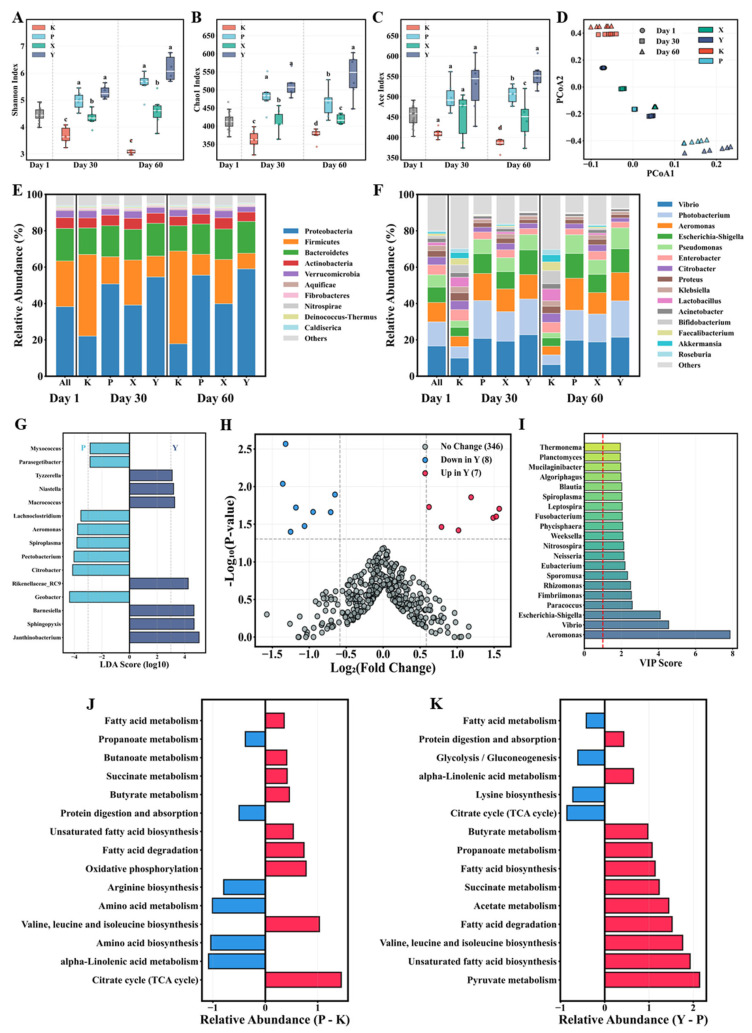
Effects of different feeds on gut microbial community structure and function in *T. septentrionalis*. (**A**–**C**) Alpha diversity indices (Shannon, Chao1, ACE); (**D**) principal coordinate analysis based on Bray–Curtis distance; (**E**) phylum-level relative abundance composition; (**F**) genus-level relative abundance (top 20 dominant genera); (**G**) LEfSe analysis-identified characteristic biomarkers in Y vs. P groups (LDA > 3.0); (**H**) volcano plot of differentially abundant genera between Y and P groups; (**I**) VIP scores at genus level (top 20 genera); (**J**,**K**) KEGG functional pathway differential enrichment analysis for Y vs. P and P vs. K comparisons. Different letters indicate significant differences among groups at the same timepoint (Tukey-adjusted, *p* < 0.05). Colors correspond to dietary treatment groups. White lines in box plots represent medians. Horizontal and vertical lines in bar plots indicate mean values and error bars, respectively. Group abbreviations are as in Figure 1.

The four feed types significantly influenced gut microbial alpha diversity (Table S8; Figure 4A–C). Shannon diversity index values in the Y group were significantly higher than all other groups (*p* < 0.05), followed sequentially by P, X, and K groups. Species richness indices Chao1 and ACE exhibited similar patterns: the Y group significantly surpassed the P, X, and K groups. Notably, all alpha diversity indices in the P group were significantly higher than those in the K group (*p* < 0.05). Principal Coordinates Analysis (PCoA) based on Bray–Curtis distances revealed significant separation of gut microbial community structures among different feed groups (Figure 4D). At Day 60, the Y and P group samples clustered relatively closely in PCoA space, while the K group samples formed a distinct separation from other groups. Time-series analysis showed that samples from each feed group displayed clear temporal gradients along the PCoA1 axis from Day 1 to Day 60.

#### 3.3.2. Gut Microbial Community Composition

Phylum-level composition (Figure 4E) revealed significant differences in gut microbiota among the four feed groups. The Y group was dominated by Firmicutes, followed by Bacteroidetes and Proteobacteria. Conversely, the K group showed a distinct distribution pattern, with Proteobacteria predominating and Firmicutes relative abundance significantly reduced. The P group displayed intermediate microbiota composition between the Y and K groups, with Firmicutes and Bacteroidetes relative abundances approaching Y group levels, while Proteobacteria were significantly lower than the K group (*p* < 0.05). Genus-level composition was more complex (Figure 4F). The Y group enriched multiple genera that were notably enriched, including *Vibrio*, *Photobacterium*, *Aeromonas*, and *Escherichia-Shigella*. It should be noted that 16S rRNA gene sequencing resolves taxonomy only to the genus level and cannot distinguish pathogenic from non-pathogenic strains within these genera. The K group exhibited distinct genus distribution: relative abundances of Enterobacter, *Lactobacillus*, and *Bifidobacterium* significantly exceeded those of other groups (*p* < 0.05). The P group resembled the Y group in most genus abundances, though *Aeromonas* abundance in the P group even surpassed that of the Y group (*p* < 0.05). VIP score analysis of the top 20 dominant genera (Figure 4I) indicated *Aeromonas* possessed the highest score, followed by *Vibrio* and *Escherichia-Shigella*, demonstrating these genera as key biomarkers distinguishing gut microbiota among different feed groups.

#### 3.3.3. Differential Microbiota Identification

LEfSe analysis (LDA ≥ 3.0, *p* < 0.05) identified seven and eight discriminative biomarkers in the Y and P groups, respectively (Figure 4G). Genera specifically enriched in the Y group included *Janthinobacterium*, *Sphingopyxis*, *Barnesiella*, and others, predominantly associated with carbohydrate degradation, short-chain fatty acid production, and immune regulation functions. The P group enriched *Geobacter*, *Citrobacter*, *Pectobacterium*, and others. Volcano plot analysis further quantified differential microbiota between Y and P groups (Figure 4H). A total of 15 significantly differential genera were identified (|Log_2_FC| > 0.58, *p* < 0.05), with seven significantly upregulated in the Y group and eight in the P group. Fold changes ranged from 0.39 to 2.97, indicating biologically significant differences in microbial composition between groups without extreme dysbiosis.

#### 3.3.4. Gut Microbial Functional Potential

Functional prediction analysis based on PICRUSt2 revealed distinct differences in the predicted functional potential of gut microbial communities among feed groups (Figure 4J,K). Comparison between Y and P groups (Figure 4J) showed the Y group showed predicted enrichment in pathways including amino acid biosynthesis, branched-chain amino acid synthesis, and fatty acid degradation (*p* < 0.05). Conversely, the P group showed predicted enrichment in pathways including protein digestion and absorption, fatty acid biosynthesis, unsaturated fatty acid synthesis, and short-chain fatty acid metabolism (butyrate, propionate, acetate) (*p* < 0.05). Functional comparison between P and K groups (Figure 4K) further suggested potential differences in predicted intestinal microbial metabolic functions between the two formulated feeds. The P group showed predicted enrichment in nutrition metabolism-related pathways, including branched-chain amino acid synthesis, fatty acid biosynthesis, and short-chain fatty acid metabolism, while the K group significantly enriched amino acid degradation and fatty acid degradation pathways (*p* < 0.05). These predictions suggest that the P group’s gut microbiota may possess enhanced predicted functional potential in branched-chain amino acid synthesis, fatty acid metabolism, and short-chain fatty acid production compared to the K group, more closely approaching the Y group’s functional characteristics. It should be noted that PICRUSt2 infers functional potential from 16S rRNA marker gene data and does not represent a direct measurement of metabolic activity. These predictions require validation by targeted approaches such as metatranscriptomics or direct metabolite quantification.

### 3.4. Effects of Feed Types on Gut Metabolic Profiles

#### 3.4.1. Overall Separation of Gut Metabolomes

**Figure 5:**
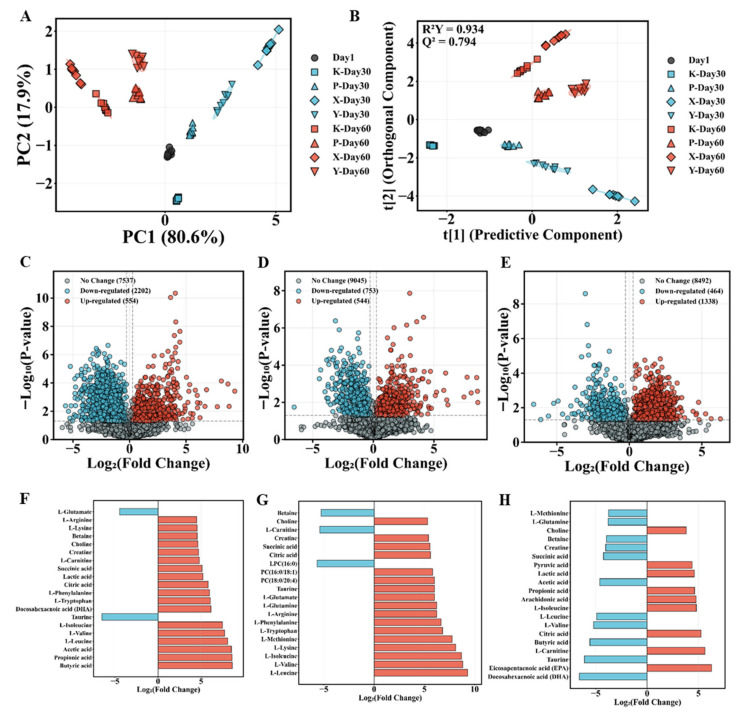
Effects of different feed types on intestinal metabolic profiles and differential metabolite identification. (**A**) Principal component analysis score plot; (**B**) OPLS-DA score plot; (**C**–**E**) volcano plots for PDX60 vs. XDX1, YDX60 vs. XDX1, and YDX60 vs. PDX60 comparisons; (**F**–**H**) top 20 differential metabolites in each comparison. Dashed lines in volcano plots indicate significance thresholds (horizontal: −log_10_(*p*-value); vertical: log_2_(fold change)). Significance levels: *p* < 0.05, *p* < 0.01, *p* < 0.001. XDX1 represents Day 1 baseline group; Day 30 and Day 60 indicate sampling at 30 and 60 days, respectively. In volcano plots (**C**–**E**), red points indicate significantly upregulated metabolites, blue points indicate significantly downregulated metabolites, and gray points represent non-significant changes. Group abbreviations are as in Figure 1.

Principal Component Analysis (PCA) and Orthogonal Partial Least Squares Discriminant Analysis (OPLS-DA) were performed on gut metabolomics data from nine experimental groups. PCA revealed clear separation among groups in principal component space. To clarify the naming convention, “XDX1” denotes the pooled Day 1 baseline samples from all groups (collected before dietary divergence), “PDX60” denotes P group samples at Day 60, and “YDX60” denotes Y group samples at Day 60. Thus, PDX60 vs. XDX1 represents the metabolic shift in the P group after 60 days of feeding relative to the common baseline; YDX60 vs. XDX1 represents the equivalent comparison for the Y group; and YDX60 vs. PDX60 captures direct metabolic differences between the Y and P groups at Day 60. PCA also showed clear separation (Figure 5A). The cumulative variance contribution of PC1 and PC2 reached 98.5%, indicating that the first two principal components sufficiently captured major information on inter-group metabolic differences. Along the PC1 axis, treatment groups at Day 30 clustered in the positive region, the Day 1 baseline group positioned near the origin, and Day 60 groups distributed in the negative region, indicating significant effects of feeding duration and different feed types on gut metabolites, with fresh fish feed and custom-formulated feed exhibiting metabolic feature similarity. The OPLS-DA multi-class discriminant model (Figure 5B) showed complete separation of experimental groups in score space, with tight within-group clustering and clear inter-group boundaries. Model performance parameters showed R^2^Y = 0.934 and Q^2^ = 0.794, confirming excellent predictive capability and robustness.

#### 3.4.2. Differential Metabolite Identification

Significantly differential metabolites for each comparison were identified through systematic volcano plot analysis (|Fold Change| > 1.2, *p* < 0.05). The PDX60 group, compared to the XDX1 baseline, detected 2955 significantly differential metabolites (Figure 5C), with 2202 upregulated and 554 downregulated. Top 20 differential metabolite analysis (Figure 5F) revealed extremely significant upregulation of free amino acids, with branched-chain amino acids (L-leucine, L-valine, L-isoleucine) exhibiting fold changes exceeding 100-fold, and significant enrichment of aromatic amino acids (L-tryptophan, L-phenylalanine) and functional amino acids (L-lysine, L-methionine, L-arginine). Additionally, key energy metabolism intermediates (citric acid, succinic acid) showed significantly elevated levels. Notably, taurine, creatine, and phospholipid metabolites exhibited significant upregulation, while lysophosphatidylcholine, L-carnitine, and betaine were significantly downregulated.

The YDX60 group, compared to the XDX1 baseline, revealed 1297 significantly differential metabolites (Figure 5D), with 544 upregulated and 753 downregulated. The lower total number of differential metabolites compared to the P group indicated more focused and specific effects of fresh fish feed on gut metabolic profiles. Top 20 differential metabolite analysis (Figure 5G) revealed extremely significant enrichment of short-chain fatty acids, with butyrate, propionate, and acetate exhibiting fold changes exceeding 350-fold. Branched-chain amino acids were likewise significantly upregulated. Notably, docosahexaenoic acid (DHA) showed significant upregulation, highly consistent with the nutritional characteristics of fresh fish ingredients rich in ω-3 polyunsaturated fatty acids. Additionally, functional metabolites including L-tryptophan, L-phenylalanine, citric acid, lactic acid, succinic acid, L-carnitine, and creatine were all significantly elevated, while taurine and L-glutamic acid exhibited significant downregulation.

Comparison between YDX60 and PDX60 groups identified 1802 significantly differential metabolites (Figure 5E), with 1338 upregulated and 464 downregulated. Top 20 differential metabolite analysis (Figure 5H) revealed key differences between the two feeds in fatty acid and energy metabolism. EPA was significantly enriched in the Y group, while DHA content was higher in the P group. Short-chain fatty acids were significantly upregulated in the Y group. Energy metabolism intermediates (citric acid, lactic acid, and pyruvic acid) and arachidonic acid were significantly elevated in the Y group, while the P group exhibited higher levels of taurine, branched-chain amino acids, succinic acid, creatine, and betaine.

#### 3.4.3. Functional Pathway Enrichment of Differential Metabolites

**Figure 6:**
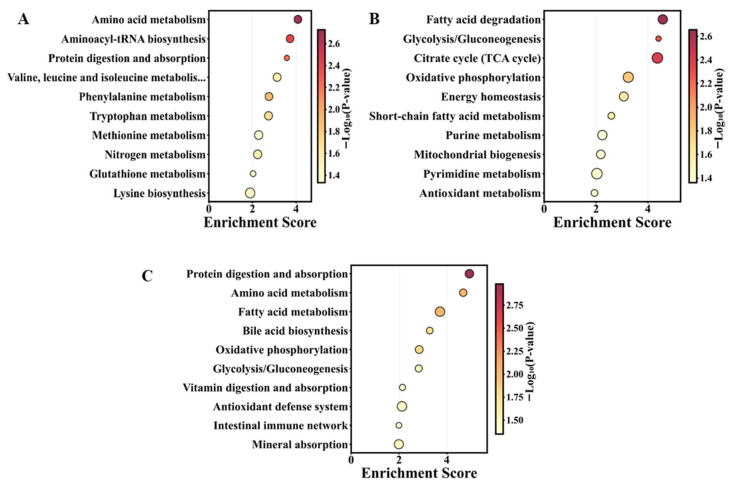
KEGG pathway enrichment analysis of differential metabolites. (**A**) PDX60 vs. XDX1; (**B**) YDX60 vs. XDX1; (**C**) YDX60 vs. PDX60. Bubble size indicates the number of enriched genes, and color represents enrichment significance (−log_10_FDR). Only pathways with FDR < 0.05 are shown.

Enrichment analysis of PDX60 vs. XDX1 (Figure 6A) indicated that differential metabolites were predominantly enriched in amino acid metabolism-related pathways, including amino acid metabolism, aminoacyl-tRNA biosynthesis, protein digestion and absorption, branched-chain amino acid metabolism (valine, leucine, isoleucine), phenylalanine metabolism, tryptophan metabolism, methionine metabolism, and nitrogen metabolism (*p* < 0.05). Additionally, glutathione metabolism and lysine biosynthesis pathways were significantly activated, indicating that custom-formulated feed significantly enhanced intestinal amino acid metabolic capacity and antioxidant defense functions.

Enrichment analysis of YDX60 vs. XDX1 (Figure 6B) showed differential metabolites were primarily enriched in energy metabolism-related pathways, including fatty acid degradation, glycolysis/gluconeogenesis, tricarboxylic acid cycle, oxidative phosphorylation, energy homeostasis, and short-chain fatty acid metabolism (*p* < 0.05). Enrichment of purine metabolism, mitochondrial biogenesis, pyrimidine metabolism, and antioxidant metabolism pathways further indicated that fresh fish feed significantly enhanced intestinal antioxidant capacity.

Enrichment analysis of YDX60 vs. PDX60 (Figure 6C) revealed key differences between the two feeds in regulating gut metabolic functions. Protein digestion and absorption, amino acid metabolism, fatty acid metabolism, bile acid biosynthesis, oxidative phosphorylation, and glycolysis/gluconeogenesis pathways were significantly enriched. Notably, pathways related to gut health and nutrient utilization, including vitamin digestion and absorption, antioxidant defense systems, intestinal immune network, and mineral absorption, were all significantly activated, indicating that fresh fish feed conferred advantages in improving intestinal barrier function, enhancing immune defense, and promoting micronutrient absorption.

### 3.5. Multi-Omics Correlation Network

**Figure 7:**
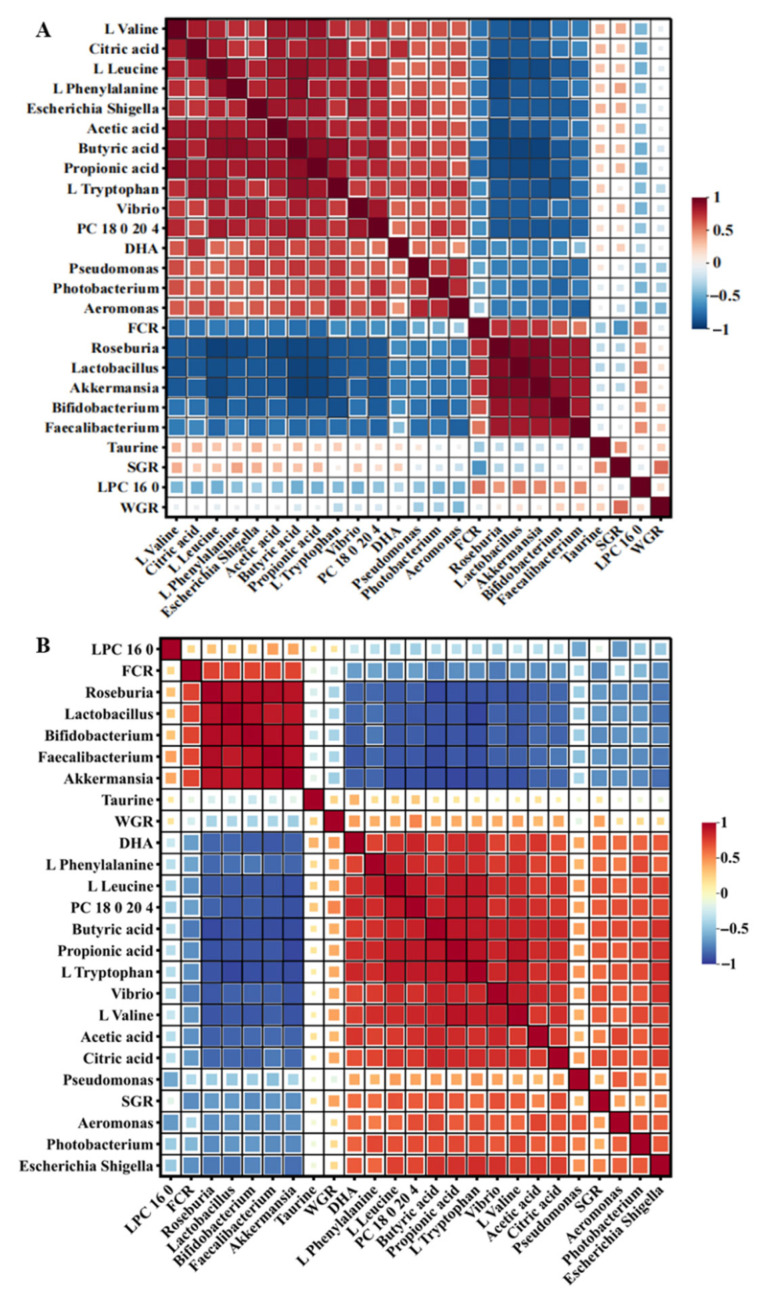
Spearman correlation networks among gut microbiota, metabolites, and growth performance. (**A**) Day 30; (**B**) Day 60. Color intensity indicates correlation coefficients (blue: negative correlation; red: positive correlation). Only correlations with |r| > 0.5 and *p* < 0.05 are shown. Square sizes are proportional to the absolute values of correlation coefficients (|r|). WGR: weight gain rate; SGR: specific growth rate; FCR: feed conversion ratio.

To explore association patterns among gut microbial communities, metabolites, and growth performance, Spearman correlation analysis was performed on multi-omics data from Day 30 and Day 60 (Figure 7). At Day 30, genera commonly associated with gut health (*Lactobacillus*, *Bifidobacterium*, *Faecalibacterium*, *Akkermansia*, *Roseburia*) formed significant negative correlation networks with potentially pathogenic genera (*Vibrio*, *Photobacterium*, *Aeromonas*, *Escherichia-Shigella*, *Pseudomonas*) (Figure 7A). Short-chain fatty acids exhibited significant positive correlations with health-associated genera and negative correlations with potentially pathogenic genera, consistent with the role of these bacteria in short-chain fatty acid synthesis. Among growth performance indicators, WGR correlated with *Aeromonas* and *Bifidobacterium*, while SGR positively correlated with butyric acid and branched-chain amino acids and negatively with *Pseudomonas*. FCR exhibited significant negative correlations with health-associated genera (*p* < 0.001) and positive correlations with potentially pathogenic genera (*p* < 0.01), indicating gut microbiota health status was closely associated with feed conversion efficiency.

By Day 60, the correlation network exhibited marked changes (Figure 7B). Microbiota–metabolite correlations strengthened overall. Association patterns between growth performance and specific microbiota-metabolite combinations shifted, with WGR showing significant positive correlations with PC 18:0/20:4, DHA, and L-valine, while SGR correlations with *Vibrio*, citric acid, and *Bifidobacterium* significantly intensified. FCR maintained significant negative correlations with all health-associated genera (*p* < 0.001) and positive correlations with potentially pathogenic genera (*p* < 0.01). Notably, lipid metabolites exhibited significantly stronger correlations with growth performance at Day 60 than at Day 30, indicating enhanced regulatory roles of lipid metabolism on growth during late culture stages. It is important to note that these correlations do not establish causality; the observed associations require experimental validation through interventional studies.

### 3.6. Growth Performance Prediction Models Based on Multi-Omics Features

**Figure 8:**
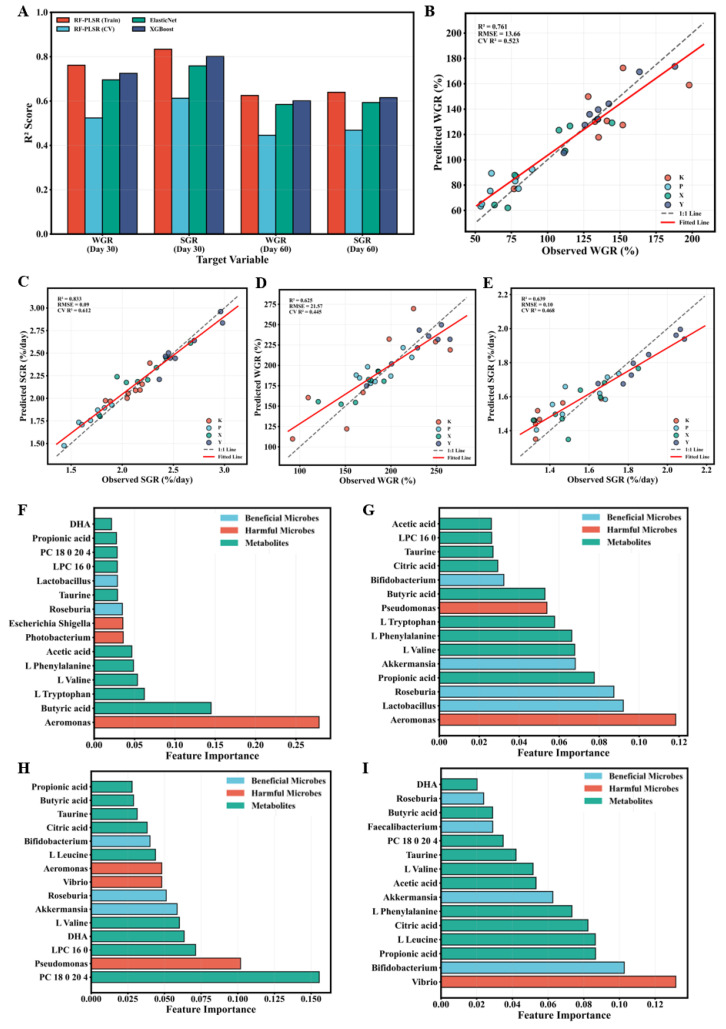
Growth performance prediction models based on multi-omics features and key feature identification. (**A**) Performance comparison of three machine learning models (RF-PLSR, ElasticNet, XGBoost); (**B**–**E**) RF-PLSR model predictions for WGR and SGR at Days 30 and 60, with solid lines representing fitted lines and dashed lines indicating 95% confidence intervals; (**F**–**I**) top 10 important features for each timepoint and metric. Feature importance calculated based on Random Forest mean impurity decrease.

Predictive models for growth performance were constructed using Random Forest feature selection combined with Partial Least Squares Regression (RF-PLSR) and compared with two conventional models, ElasticNet and XGBoost (Figure 8A). The RF-PLSR model showed the best exploratory predictive performance across all target variables. At Day 30, the RF-PLSR model achieved a training R^2^ of 0.761 and an 8-fold cross-validation R^2^ of 0.523 for WGR (Figure 8B); for SGR, the training R^2^ was 0.833 with a cross-validation R^2^ of 0.612 (Figure 8C). In contrast, ElasticNet models achieved cross-validation R^2^ of 0.486 (WGR) and 0.571 (SGR), while XGBoost models achieved 0.512 (WGR) and 0.594 (SGR), all lower than RF-PLSR models. By Day 60, model prediction performance declined somewhat. The RF-PLSR model achieved cross-validation R^2^ of 0.445 for WGR (Figure 8D) and 0.468 for SGR (Figure 8E), but still significantly outperformed ElasticNet and XGBoost baseline models.

Random Forest feature importance analysis identified key multi-omics features with the greatest contributions to growth performance prediction. At Day 30, the top five features for WGR prediction were as follows: *Aeromonas*, butyric acid, L-tryptophan, L-valine, and L-phenylalanine (Figure 8F); for SGR prediction, *Aeromonas*, *Lactobacillus*, *Roseburia*, propionic acid, and *Akkermansia* (Figure 8G). By Day 60, feature importance rankings shifted significantly. For WGR prediction, the top five features became PC 18:0/20:4, *Pseudomonas*, LPC 16:0, DHA, and L-valine (Figure 8H), with cumulative importance of lipid metabolites increasing from 10.2% at Day 30 to 36.8%; for SGR prediction, the top five features were *Vibrio*, *Bifidobacterium*, propionic acid, L-leucine, and citric acid (Figure 8I).

To further assess model generalizability, a hold-out validation set (20% of samples, *n* = 7 at Day 30 and *n* = 10 at Day 60) was evaluated using the trained RF-PLSR models. On the hold-out set, the model achieved R^2^ values of 0.487 (WGR, Day 30), 0.521 (SGR, Day 30), 0.402 (WGR, Day 60), and 0.418 (SGR, Day 60), with corresponding RMSE values of 12.83, 0.38, 18.45, and 0.29, respectively (Table S10; Figure S1). While these values are lower than the cross-validation results, they remain within an acceptable range for exploratory biological models and indicate that the models capture genuine biological signals rather than merely fitting noise. Nevertheless, external validation on independent cohorts is warranted to confirm the robustness of these predictive features.

## 4. Discussion

The present findings suggest that dietary composition is associated with distinct differences in gut microbiota composition and metabolic profiles in *T. septentrionalis*. The fresh fish group showed notable differences in growth performance, intestinal microbiota, and metabolism, while the custom-formulated feed group achieved levels comparable to fresh fish across most indicators through optimized nutrient composition, substantially outperforming the commercial pelleted feed group.

### 4.1. Differential Effects of Feed Types on Growth Performance and Nutritional Basis

The fresh fish group significantly outperformed all other groups in final body weight, WGR, and SGR, with an FCR of only 1.14. This difference may be attributed to the abundance of easily digestible myofibrillar proteins, high EPA and DHA content, and substantial free amino acids and bioactive peptides in small yellow croaker muscle [27], resulting in apparent digestibility significantly higher than formulated feeds, as similarly reported in grouper (*Epinephelus* spp.) and large yellow croaker (*Larimichthys crocea*) [10,11]. Although the P group exhibited lower WGR than the Y group, it still showed notable advantages over the K group, attributable to 40% high-quality fishmeal and 5% fish oil supplementation.

From an economic perspective, while the 40% fishmeal inclusion in the P group formulation increases raw material costs relative to the commercial K group diet (estimated feed cost: P group ~¥12,800/ton vs. K group ~¥8500/ton based on current fishmeal prices of approximately ¥12,000–15,000/ton; [2,3]), the superior FCR achieved by the P group (1.40 vs. 1.70 at Day 60 on a dry-matter basis) partially offsets this cost difference. The estimated feed cost per kilogram of weight gain was ¥17.92/kg for P vs. ¥14.45/kg for K at Day 60 (Table S9). However, the P group also achieved significantly higher muscle protein content and essential amino acid levels, which may translate to premium market value for this species. Future optimization strategies could include partial replacement of fishmeal with sustainable alternative protein sources such as insect meal (Hermetia illucens), single-cell proteins, or fermented soybean meal to reduce costs while maintaining the nutritional advantages observed in the high-fishmeal formulation [4,9].

Fishmeal is recognized as an irreplaceable protein source in marine fish formulated feeds due to its amino acid composition highly matched to fish muscle proteins, balanced essential amino acids, and enrichment in functional nitrogenous compounds such as taurine [2,4,34]. The P group’s superior performance compared to the K group can be attributed to its higher dry matter-based protein content (62.7% vs. 58.3%) and elevated lipid levels (15.8% vs. 12.5%), providing greater energy density and essential fatty acids. The poorest growth performance in the K group likely relates to lower protein quality and antinutritional factors in plant proteins, including trypsin inhibitors, phytic acid, and tannins, which not only reduce host nutrient digestibility but may also indirectly shape gut microbial community structure through altered intestinal pH [35,36].

### 4.2. Patterns and Potential Implications of Muscle Nutritional Quality Differences

The X and P groups achieved muscle protein contents of 19.90% and 20.30% at Day 60, significantly higher than the fastest-growing Y group, indicating a trade-off between rapid growth and protein deposition rate. In Atlantic salmon (*Salmo salar*) and rainbow trout (*Oncorhynchus mykiss*), rapidly growing individuals often exhibit relatively higher muscle moisture content, attributed to extracellular matrix expansion and tissue fluid accumulation during rapid muscle fiber hyperplasia [27,29]. Notably, muscle fatty acid composition differed markedly. The Y and X groups reached EPA + DHA contents of 32.81% and 30.70% at Day 60, far exceeding the K group. Based on the WHO/FAO recommended daily EPA + DHA intake of 250–500 mg, consuming only 200–250 g of Y group *T. septentrionalis* would meet this recommendation [11,27]. The Y group achieved an n-3/n-6 ratio of 3.69, significantly higher than the K group, holding important nutritional significance for addressing the excessive n-6/n-3 ratio in modern human diets [35]. The P and X groups exhibited significantly elevated total free amino acid content at Day 60, particularly umami amino acids such as glutamic acid, which can substantially enhance muscle sensory quality [34,35], providing scientific evidence for product positioning and value enhancement.

### 4.3. Feed-Associated Differences in Gut Microbiota Composition

A notable observation in this study is the marked increase in gut microbial diversity in the Y and P groups compared to the K group. Enhanced diversity is often considered indicative of more diverse gut ecosystems, though the relationship between diversity and function is complex [36]. Fresh fish feeds rich in diverse natural proteins, polyunsaturated fatty acids, and bioactive peptides provide diversified nutritional substrates for gut microbiota. PCoA analysis revealed a clear separation of the K group samples from other groups, indicating feed type as a primary driver shaping gut microbial structure. At the phylum level, Firmicutes’ relative abundance in the Y and P groups reached 45.3% and 41.7%, significantly higher than 28.5% in the K group, while Proteobacteria exhibited the opposite trend. This pattern of elevated Firmicutes and reduced Proteobacteria is recognized as a healthy gut signature [36]. Firmicutes harbor abundant short-chain fatty acid-producing bacteria that ferment recalcitrant substrates to generate butyrate, propionate, and acetate, providing the primary energy source for intestinal epithelial cells [34,37]. Conversely, Proteobacteria relative abundance exceeded 50% in the K group, with enrichment of potential pathogens such as *Vibrio* and *Aeromonas* potentially increasing intestinal infection risk and chronic inflammatory burden [35].

Notably, among the genera identified by LEfSe analysis as differentially enriched, *Lactobacillus* was markedly enriched in the Y and P groups. Lactobacilli exert probiotic effects through multiple mechanisms, including lactic acid production to lower intestinal pH, bacteriocin production to inhibit pathogens, competitive occupation of adhesion sites, and host immune system modulation [27,38,39]. *Faecalibacterium* abundance in the Y group increased 4.9-fold over the K group. *Faecalibacterium prausnitzii* is a major butyrate producer whose butyrate serves as the preferred energy substrate for intestinal epithelial cells while functioning as an HDAC inhibitor to upregulate intestinal barrier genes and triggering anti-inflammatory responses through GPR109A and GPR43 receptor activation [34,36]. *Akkermansia* abundance reached 4.2% in the Y group, a 10.5-fold increase over the K group. *Akkermansia muciniphila* obligately degrades intestinal mucin, with its outer membrane protein enhancing intestinal tight junctions via Toll-like receptor 2 in mammals [37,38]. The significant *Akkermansia* enrichment in the Y group may relate to higher natural mucin and glycoprotein content in fresh fish ingredients.

### 4.4. Intestinal Metabolic Profiles and Potential Regulatory Pathways

The distinct separation of gut metabolomic profiles among feeding groups highlights the substantial differences in intestinal metabolite profiles associated with dietary composition. The PC1 axis primarily reflected feeding duration effects, indicating progressive adjustment of intestinal metabolic status with extended feeding cycles [27]. The Y and P groups exhibited relatively close distributions in metabolic space, yet 1802 significantly different metabolites were identified between them, with upregulated metabolites comprising 74.3%, indicating fresh fish feed advantages in elevating intestinal metabolic activity [29]. Short-chain fatty acid content in the Y group significantly exceeded that of the other groups. Short-chain fatty acids, primarily produced by gut microbial fermentation, serve as preferred energy substrates for intestinal epithelial cells [18,36]. Butyrate not only provides approximately 70% of colonocyte energy requirements but also exhibits anti-inflammatory properties, regulates gene expression, and maintains intestinal barrier integrity [18]. The substantial butyrate elevation in the Y group derives from fresh fish feed-mediated gut microbial community shaping that enriched butyrate-producing bacteria, while high digestibility of fresh fish protein provided quality fermentation substrates for short-chain fatty acid-producing bacteria [34,35]. Based on mammalian and fish studies, butyrate promotes growth by serving as the primary energy source for intestinal epithelial cells, upregulating barrier gene expression as an HDAC inhibitor, and triggering anti-inflammatory signals through GPR109A and GPR43 receptor activation [36,40].

Branched-chain amino acids displayed significant upregulation in both P and Y groups, with slightly greater magnitude in the P group. Branched-chain amino acids are recognized as important protein synthesis precursors and have been reported to influence cell growth and metabolism via the mTOR signaling pathway in other species [41]. In model organisms, leucine activates mTORC1 via the Sestrin2 sensor and Rag GTPases, subsequently phosphorylating downstream effectors to promote translation initiation and elongation [42,43]. The P group muscle crude protein content reached 20.30%, correlating with elevated branched-chain amino acid levels, suggesting the branched-chain amino acid-mTOR axis may participate in regulating muscle protein synthesis [40,41]. Additionally, PC (18:0/20:4) containing arachidonic acid serves as a precursor for prostaglandin synthesis and other inflammatory mediators [35]. Lysophosphatidylcholine functions as a membrane phospholipid turnover intermediate and can participate in GLP-1 secretion and glucose homeostasis regulation via GPR119 and S1P receptors [36]. The highly significant DHA elevation in the Y group enables greater DHA incorporation into cell membranes, enhancing membrane fluidity and functional activity, while DHA-derived specialized pro-resolving mediators possess potent anti-inflammatory and tissue repair-promoting properties [37,38]. KEGG pathway enrichment analysis revealed significant Y group enrichment in core energy metabolism pathways, including fatty acid degradation, glycolysis/gluconeogenesis, TCA cycle, and oxidative phosphorylation, indicating fresh fish feed provides robust energy support for intestinal epithelial cells through optimized intestinal energy metabolic pathways [30,33].

### 4.5. Multi-Omics Integration: Associations Between Gut Microbiota, Metabolites, and Host Growth

The reproducibility of positive correlations between health-associated genera and short-chain fatty acids across both Day 30 and Day 60, with enhanced effects at Day 60. Conversely, genera containing potentially pathogenic species exhibited negative correlations with growth indices [27]. FCR showed significant negative correlations with health-associated genera and positive correlations with potential pathogenic genera, indicating that gut microecological health status closely relates to feed conversion efficiency. Healthy gut microecology may improve nutrient digestibility and absorption efficiency through short-chain fatty acid-derived energy provision, B-vitamin and amino acid synthesis, and intestinal barrier integrity maintenance, while pathogen proliferation induces chronic inflammation and diverts energy toward immune responses [14,29]. From Day 30 to Day 60, correlations between lipid metabolites and growth performance intensified significantly, indicating enhanced lipid metabolism regulatory roles in later-stage growth [27]. This likely relates to increased fish demand for lipids as efficient energy sources and bioactive molecules during rapid growth phases.

From a methodological perspective, the RF-PLSR integrated model suggested that gut microbial and metabolite features may serve as candidate predictors of growth performance, with cross-validation R^2^ reaching 0.523–0.612 at Day 30 and 0.445–0.468 at Day 60. Considering the small sample size and inherent biological system complexity, these levels are acceptable and significantly outperform two common baseline models. Feature importance analysis revealed that at Day 30, the high importance of short-chain fatty acids and their producing bacteria suggested short-chain fatty acids as candidate signaling molecules in the “gut-brain-growth axis” [44]. The high feature importance of *Aeromonas* suggested its potential as a candidate “negative growth biomarker,” with these opportunistic pathogens impairing host health through lipopolysaccharide-triggered chronic inflammation and competitive inhibition of commensal bacterial colonization [36,37]. By Day 60, lipid metabolites emerged as the most important predictive features, reflecting increased lipid demand during rapid fish growth [38].

### 4.6. Study Limitations, Application Insights, and Future Perspectives

This study tracked only a 60-day culture period, covering the rapid growth phase of juvenile *T. septentrionalis* but failing to observe long-term feed effects during sexual maturation. In species such as Atlantic salmon (*Salmo salar*) and rainbow trout (*Oncorhynchus mykiss*), feed response patterns in early culture stages may differ significantly from subsequent growth phases [27,29]. Sample size limitations may have constrained the machine learning model’s generalizability. To mitigate this, bootstrap resampling (1000 iterations) was used as the primary data augmentation strategy during model training, and a stratified hold-out set (20%) provided independent validation (see Section 2.9 and Table S10). Nevertheless, the limited sample size and the exploratory nature of these models necessitate external validation on independent cohorts from different facilities and seasons before the identified predictive features can be considered robust biomarker capability. Although data augmentation strategies partially mitigated small sample issues, this approach only generates new samples within existing sample distributions and cannot capture unsampled biological variation. 16S rRNA gene sequencing provides only genus-level taxonomic information; metagenomic sequencing would enable more precise dissection of functional gene composition and actual metabolic pathway activity [14]. Future research should validate causal relationships through interventional experiments. Probiotic supplementation trials can verify growth-promoting effects of *Lactobacillus* plantarum and *Faecalibacterium prausnitzii*, metabolite gavage experiments can confirm physiological functions of butyrate and branched-chain amino acids, and microbiota transplantation experiments can observe growth performance and metabolic feature shifts [34,35]. Integrating host transcriptomic and proteomic data can deepen understanding of gut microbiota-host interaction mechanisms at gene expression and protein translation levels [36]. Expanding sample size to at least 100–150 fish across multiple batches and seasons will facilitate establishing predictive models with cross-scenario generalization capability [37], providing strategies for precision development of *T. septentrionalis* formulated feeds.

Additionally, although each treatment was replicated with three independent tanks (the true experimental units for feed effect comparisons), this design yields *n* = 3 per treatment for tank-level statistical inference. While individual fish measurements (*n* = 25 per tank) were used for data visualization to show biological variability, all statistical tests for feed effects were conducted using tank means to avoid pseudoreplication. Future studies should consider increasing the number of replicate tanks to enhance statistical power for detecting treatment differences.

## 5. Conclusions

This study identified associations between different feed types and *T. septentrionalis* growth performance, gut microbiota composition, and intestinal metabolite profiles in *T. septentrionalis* through integrated multi-omics profiling. The fresh fish group significantly outperformed other groups in growth performance, feed conversion efficiency, and muscle EPA + DHA content, primarily through shaping healthy gut microbial structure (high Firmicutes abundance, low Proteobacteria abundance) and markedly elevated short-chain fatty acid metabolism. The custom-formulated feed group, through optimized fishmeal and fish oil ratios, achieved levels comparable to the fresh fish group across most indicators, including gut microbial diversity, branched-chain amino acid metabolism, and muscle protein deposition, significantly surpassing the commercial pelleted feed group. Multi-omics predictive models based on Random Forest feature selection identified key microbial-metabolite features, including *Aeromonas*, short-chain fatty acids, branched-chain amino acids, and phospholipids, providing quantifiable biomarkers for growth performance differences. Future research should integrate metagenomic sequencing to precisely quantify metabolic pathway functional potential, combine host transcriptomic data to reveal molecular regulatory networks of the gut-liver-muscle axis, and validate causal relationships of key features through probiotic supplementation, metabolite gavage, and microbiota transplantation experiments, thereby providing scientific evidence for precision feed development in intensive *T. septentrionalis* aquaculture and promoting sustainable industry development.

## Data Availability

The original contributions presented in this study are included in the article/Supplementary Materials. Further inquiries can be directed to the corresponding authors.

## Supplementary Materials

The following supporting information can be downloaded at: https://www.mdpi.com/article/10.3390/ani16091312/s1, Table S1: Feed ingredient formulation of pelleted formulated feeds; Table S2: Proximate composition of the four experimental feeds; Table S3: Growth performance and morphometric indices of *Thamnaconus septentrionalis* fed different diets; Table S4: Derived growth performance indicators of *Thamnaconus septentrionalis* fed different diets; Table S5: Proximate composition of muscle in *Thamnaconus septentrionalis* fed different diets across culture periods; Table S6: Free amino acid composition of muscle in *Thamnaconus septentrionalis* fed different diets across culture periods; Table S7: Fatty acid composition of muscle in *Thamnaconus septentrionalis* fed different diets across culture periods; Table S8: Alpha diversity indices of gut microbiota in *Thamnaconus septentrionalis* fed different diets; Table S9: Economic cost analysis of the four experimental feeds for *Thamnaconus septentrionalis* culture; Table S10: Hold-out validation performance of RF-PLSR growth prediction models; Figure S1: Hold-out validation of RF-PLSR growth prediction models.
