# Supplementary material for: Dietary Modulation of Gut Microbiota and Metabolome Shapes Growth Performance in Thamnaconus septentrionalis
Source: Animals (Basel). 2026 Apr 24;16(9):1312. doi: 10.3390/ani16091312 (PMC13162955; doi:10.3390/ani16091312)
Supplement: Supplementary file 1 [file animals-16-01312-s001.zip › animals-4229731-supplementary.pdf]

Table S1. Feed ingredient formulation of pelleted formulated feeds (as formulated basis, %).

Ingredient	K (%)	P (%)
Fishmeal	22	40
Soybean meal	30	15
Cottonseed	10	0
Wheat flour	20	20
Corn starch	8	10
Fish oil	1.5	5
Soybean	3	2
$Ca(H_2PO_4)_2$	2	2.5
Vitamin premix	1.5	2
Mineral premix	1	1.5
Choline chloride	0.5	0.5
Binder (CMC)	0.3	1
Antioxidant (BHT)	0.2	0.5
Total	100	100

.

Note: Both K and P groups were manufactured as extruded pellets using identical processing parameters: extrusion temperature 110-120°C, moisture content during extrusion 25-30%, final pellet moisture 10-12%, pellet diameter 3.0 mm, expansion coefficient 2.5-3.0. Groups X (frozen shrimp slices) and Y (fresh fish chunks) were prepared as described in Section 2.3 of the main text.

Table S2. Proximate composition of the four experimental feeds (on a dry matter basis, %)

Group	Crude Protein	Crude Lipid	Ash	Crude Fiber
K	58.3±0.9	12.5±0.6	14.2±0.7	4.5±0.4
P	62.7±0.8	15.8±0.7	12.3±0.6	3.2±0.3
X	68.5±1.2	8.2±0.5	18.9±0.8	-
Y	72.1±1.1	6.5±0.4	15.4±0.7	-

Note: All values are expressed on a dry matter basis to enable direct comparison between formulated feeds (K, P) and fresh feeds (X, Y) with substantially different moisture contents. Original moisture contents were: K (10.5%), P (10.8%), X (76.2%), and Y (78.5%).

Table S3. Growth performance and morphometric indices of Thamnaconus septentrionalis fed different diets

Indicator	Body Weight (g)	Total Length (mm)	Body Length (mm)	Body Height (mm)	Body Thickness (mm)	Head Length (mm)	Eye Diameter (mm)	Preorbital Length (mm)	Viscera Weight (g)	Liver Weight (g)
K-Day 1	28.99±1.93a	129.37±2.19a	118.06±2.16a	41.51±1.04a	16.33±0.36a	33.82±0.57a	10.10±0.13a	28.54±0.56a	5.26±0.35a	3.43±0.20a
P-Day 1	34.26±1.89a	132.88±1.92a	113.67±2.27a	43.69±1.15a	15.60±0.38a	33.93±0.67a	9.31±0.17b	27.27±0.52a	5.24±0.29a	3.37±0.22a
X-Day 1	34.22±2.14a	128.89±2.89a	113.14±1.81a	41.12±1.01a	15.64±0.42a	34.37±0.53a	9.58±0.18b	27.86±0.50a	4.97±0.34a	3.81±0.17a
Y-Day 1	30.75±1.86a	128.75±2.61a	117.41±1.83a	44.10±1.10a	15.23±0.37a	34.43±0.62a	9.62±0.15b	28.90±0.74a	5.14±0.27a	3.36±0.25a
K-Day 30	50.86±0.86c	150.84±2.67b	133.89±1.98a	47.79±0.71c	17.45±0.22d	40.25±0.52a	10.08±0.12a	34.87±0.48a	8.90±0.17b	4.84±0.08c
P-Day 30	51.74±0.58c	145.02±2.20b	132.75±1.21a	49.96±0.61b	18.77±0.30c	38.97±0.52a	10.07±0.13a	32.72±0.44b	9.22±0.13b	5.81±0.06b
X-Day 30	61.58±0.80b	154.08±1.42b	138.00±2.06a	51.62±0.72b	19.62±0.29b	39.44±0.47a	10.09±0.14a	34.29±0.46a	9.51±0.13b	5.75±0.07b
Y-Day 30	64.66±0.71a	160.24±1.89a	142.77±2.09a	54.11±0.62a	20.66±0.27a	41.07±0.54a	10.21±0.14a	34.96±0.42a	11.93±0.14a	7.42±0.07a
K-Day 60	62.05±1.05c	173.34±2.62b	148.09±2.42b	54.57±0.75b	18.97±0.33d	47.25±0.57a	10.08±0.11b	42.35±0.72a	12.80±0.19b	6.16±0.06d
P-Day 60	87.24±1.32a	158.52±3.11c	152.69±1.38b	55.46±0.86b	22.06±0.27c	43.95±0.59b	10.91±0.17a	37.58±0.63b	13.27±0.17b	8.46±0.16b
X-Day 60	81.05±1.21b	175.19±2.76b	162.92±2.49a	62.63±1.11a	23.47±0.36b	45.18±0.58b	10.69±0.15a	41.15±0.79a	13.79±0.25b	7.76±0.11c
Y-Day 60	87.23±1.33a	194.92±3.00a	168.96±2.34a	64.80±0.90a	26.25±0.48a	48.41±0.77a	10.91±0.15a	41.09±0.51a	19.49±0.22a	12.07±0.17a

Table S4. Derived growth performance indicators of Thamnaconus septentrionalis fed different diets

Indicator	Weight Gain Rate (%)	Specific Growth Rate (%/day)	Hepatosomatic Index (%)	Viscerosomatic Index (%)	Condition Factor (g/cm³)	Feed Conversion Ratio
**K-Day 30**	124.33±33.59a	2.17±0.29a	9.61±0.23b	17.62±0.41a	2.19±0.10a	1.78±0.02a
**P-Day 30**	72.88±15.12a	1.56±0.23a	11.26±0.17a	17.90±0.35a	2.24±0.06a	1.47±0.01b
**X-Day 30**	113.49±22.70a	2.19±0.25a	9.40±0.19b	15.54±0.31b	2.43±0.10a	1.39±0.01c
**Y-Day 30**	146.49±22.43a	2.70±0.25a	11.51±0.13a	18.52±0.29a	2.31±0.11a	1.20±0.01d
**K-Day 60**	180.73±46.96a	1.41±0.16a	10.01±0.18b	20.78±0.42b	2.00±0.10b	1.70±0.02a
**P-Day 60**	189.39±24.00a	1.65±0.11a	9.74±0.21b	15.33±0.32d	2.48±0.07a	1.40±0.01b
**X-Day 60**	181.99±31.29a	1.55±0.13a	9.64±0.21b	17.12±0.39c	1.94±0.09b	1.31±0.01c
**Y-Day 60**	232.10±29.96a	1.84±0.12a	13.92±0.27a	22.47±0.35a	1.87±0.09b	1.14±0.01d

Note: All values are presented as mean ± SEM (standard error of the mean). Different superscript letters within the same row indicate significant differences among groups ($P < 0.05$, Tukey's HSD test with Benjamini-Hochberg FDR correction). Feed conversion ratio (FCR) values were calculated on a dry-matter basis (FCR_DM = dry matter feed intake / wet weight gain) to enable valid comparison across feed types with different moisture contents (K: 10.5%, P: 10.8%, X: 76.2%, Y: 78.5%).

Table S5. Proximate composition of muscle in Thamnaconus septentrionalis fed different diets across culture periods

Indicator	Day 1	Day 30 - K	Day 30 - P	Day 30 - X	Day 30 - Y	Day 60 - K	Day 60 - P	Day 60 - X	Day 60 - Y
Moisture (%)	77.87 ± 0.10	78.53 ± 0.07a	78.15 ± 0.11a	77.93 ± 0.10a	78.35 ± 0.12a	78.34 ± 0.13a	77.34 ± 0.10b	77.54 ± 0.08b	78.28 ± 0.06a
Protein (%)	19.10 ± 0.03	19.79 ± 0.02c	20.09 ± 0.02b	21.29 ± 0.03a	18.55 ± 0.02d	18.01 ± 0.03d	20.33 ± 0.02a	19.91 ± 0.03b	19.10 ± 0.02c
Lipid (%)	0.80 ± 0.00	0.90 ± 0.00a	0.80 ± 0.00b	0.90 ± 0.00a	0.80 ± 0.00b	0.70 ± 0.00a	0.60 ± 0.00b	0.70 ± 0.00a	0.70 ± 0.00a
Total Free Amino Acids (g/100g)	18.315 ± 0.008	18.840 ± 0.007c	19.038 ± 0.008b	20.460 ± 0.008a	17.405 ± 0.007d	17.322 ± 0.006d	19.149 ± 0.008a	19.053 ± 0.006b	18.153 ± 0.004c
Essential Amino Acids (g/100g)	8.113 ± 0.005	8.226 ± 0.003c	8.438 ± 0.005b	9.104 ± 0.005a	7.690 ± 0.004d	7.571 ± 0.003d	8.418 ± 0.003a	8.261 ± 0.005b	7.906 ± 0.004c
Non-Essential Amino Acids (g/100g)	10.202 ± 0.004	10.614 ± 0.007b	10.599 ± 0.005b	11.356 ± 0.008a	9.715 ± 0.005c	9.751 ± 0.004d	10.731 ± 0.006b	10.792 ± 0.004a	10.247 ± 0.004c
Saturated Fatty Acids (%)	32.109 ± 0.032	29.164 ± 0.026a	29.064 ± 0.027b	27.585 ± 0.028d	28.897 ± 0.019c	28.389 ± 0.016c	28.993 ± 0.021b	30.054 ± 0.022a	27.686 ± 0.025d
Monounsaturated Fatty Acids (%)	25.704 ± 0.018	20.602 ± 0.038c	23.640 ± 0.033a	16.675 ± 0.020d	22.889 ± 0.027b	22.031 ± 0.024a	19.775 ± 0.021c	18.850 ± 0.017d	21.240 ± 0.015b
Polyunsaturated Fatty Acids (%)	32.654 ± 0.022	40.932 ± 0.028b	38.939 ± 0.033d	46.470 ± 0.025a	39.168 ± 0.022c	40.419 ± 0.034d	43.417 ± 0.032a	41.976 ± 0.026c	42.115 ± 0.037b
EPA+DHA (%)	18.646 ± 0.020	17.453 ± 0.019d	25.712 ± 0.029c	33.720 ± 0.023a	26.509 ± 0.019b	27.441 ± 0.033d	30.623 ± 0.029c	30.750 ± 0.025b	32.751 ± 0.036a
n-3/n-6 Ratio	1.610 ± 0.003	0.877 ± 0.001d	2.248 ± 0.004c	2.916 ± 0.003a	2.412 ± 0.003b	2.393 ± 0.004d	2.699 ± 0.003c	3.191 ± 0.004b	3.960 ± 0.005a

Table S6. Free amino acid composition of muscle in Thamnaconus septentrionalis fed different diets across culture periods

Amino Acid	Day 1	Day 30 - K	Day 30 - P	Day 30 - X	Day 30 - Y	Day 60 - K	Day 60 - P	Day 60 - X	Day 60 - Y
Asp	1.917 ± 0.002	1.993 ± 0.003b	1.975 ± 0.003c	2.139 ± 0.004a	1.808 ± 0.003d	1.820 ± 0.002d	2.023 ± 0.002a	1.980 ± 0.002b	1.910 ± 0.003c
Thr	0.878 ± 0.001	0.889 ± 0.001c	0.911 ± 0.001b	0.990 ± 0.002a	0.830 ± 0.001d	0.822 ± 0.001d	0.909 ± 0.001a	0.889 ± 0.001b	0.862 ± 0.001c
Ser	0.732 ± 0.001	0.750 ± 0.001c	0.759 ± 0.001b	0.819 ± 0.001a	0.699 ± 0.001d	0.698 ± 0.001d	0.768 ± 0.001b	0.779 ± 0.001a	0.740 ± 0.001c
Glu	3.060 ± 0.003	3.185 ± 0.006b	3.142 ± 0.003c	3.355 ± 0.005a	2.814 ± 0.003d	2.880 ± 0.003c	3.200 ± 0.004a	3.203 ± 0.003a	2.984 ± 0.003b
Gly	0.862 ± 0.001	0.877 ± 0.001c	0.902 ± 0.002b	0.979 ± 0.001a	0.880 ± 0.001c	0.840 ± 0.001d	0.920 ± 0.001c	0.961 ± 0.001a	0.930 ± 0.001b
Ala	1.132 ± 0.001	1.191 ± 0.002b	1.191 ± 0.001b	1.283 ± 0.002a	1.109 ± 0.002c	1.102 ± 0.001d	1.210 ± 0.002a	1.199 ± 0.001b	1.159 ± 0.002c
Cys	0.120 ± 0.000	0.140 ± 0.000b	0.150 ± 0.000a	0.150 ± 0.000a	0.130 ± 0.000c	0.130 ± 0.000c	0.130 ± 0.000c	0.150 ± 0.000a	0.140 ± 0.000b
Val	1.060 ± 0.002	1.091 ± 0.002c	1.113 ± 0.002b	1.192 ± 0.002a	1.031 ± 0.001d	1.010 ± 0.001d	1.117 ± 0.002a	1.089 ± 0.002b	1.069 ± 0.001c
Met	0.500 ± 0.001	0.549 ± 0.001c	0.571 ± 0.001b	0.611 ± 0.001a	0.500 ± 0.001d	0.500 ± 0.000d	0.559 ± 0.001b	0.569 ± 0.001a	0.520 ± 0.001c
Ile	0.941 ± 0.001	0.982 ± 0.001c	0.989 ± 0.001b	1.070 ± 0.002a	0.907 ± 0.002d	0.897 ± 0.001d	1.009 ± 0.001a	0.976 ± 0.001b	0.940 ± 0.001c
Leu	1.563 ± 0.003	1.630 ± 0.002c	1.647 ± 0.002b	1.766 ± 0.002a	1.491 ± 0.002d	1.472 ± 0.002d	1.642 ± 0.001a	1.620 ± 0.002b	1.524 ± 0.002c
Tyr	0.570 ± 0.001	0.671 ± 0.001c	0.690 ± 0.001b	0.699 ± 0.001a	0.620 ± 0.000d	0.580 ± 0.001c	0.640 ± 0.001a	0.641 ± 0.001a	0.592 ± 0.001b
Phe	0.760 ± 0.001	0.769 ± 0.001c	0.790 ± 0.001b	0.860 ± 0.001a	0.740 ± 0.000d	0.740 ± 0.001d	0.811 ± 0.001a	0.789 ± 0.001b	0.770 ± 0.001c
Lys	1.972 ± 0.004	1.867 ± 0.002c	1.928 ± 0.002b	2.085 ± 0.003a	1.762 ± 0.003d	1.711 ± 0.001d	1.909 ± 0.002a	1.879 ± 0.003b	1.781 ± 0.002c
His	0.439 ± 0.001	0.450 ± 0.000c	0.490 ± 0.000b	0.530 ± 0.001a	0.429 ± 0.001d	0.420 ± 0.001d	0.461 ± 0.000a	0.450 ± 0.001b	0.440 ± 0.000c
Arg	1.200 ± 0.002	1.217 ± 0.002c	1.229 ± 0.001b	1.342 ± 0.002a	1.117 ± 0.002d	1.140 ± 0.001d	1.241 ± 0.001b	1.260 ± 0.001a	1.191 ± 0.002c
Pro	0.610 ± 0.001	0.588 ± 0.001a	0.560 ± 0.001b	0.591 ± 0.001a	0.540 ± 0.001c	0.560 ± 0.001c	0.599 ± 0.001b	0.619 ± 0.001a	0.601 ± 0.001b

Table S7. Fatty acid composition of muscle in Thamnaconus septentrionalis fed different diets across culture periods

Fatty Acid	Day 1	Day 30 - K	Day 30 - P	Day 30 - X	Day 30 - Y	Day 60 - K	Day 60 - P	Day 60 - X	Day 60 - Y
C14:0	1.683 ± 0.002	0.927 ± 0.002d	1.678 ± 0.002a	1.120 ± 0.001b	1.040 ± 0.001c	0.952 ± 0.002d	1.009 ± 0.002b	1.140 ± 0.002a	0.966 ± 0.001c
C15:0	0.457 ± 0.001	0.364 ± 0.001c	0.390 ± 0.001b	0.330 ± 0.000d	0.413 ± 0.001a	0.394 ± 0.001b	0.395 ± 0.001b	0.491 ± 0.001a	0.377 ± 0.001c
C16:0	20.661 ± 0.029	18.217 ± 0.027a	17.801 ± 0.026b	16.904 ± 0.026c	15.716 ± 0.013d	15.369 ± 0.018c	16.202 ± 0.020b	16.801 ± 0.022a	14.007 ± 0.025d
C16:1n7c	5.773 ± 0.007	2.581 ± 0.005d	4.583 ± 0.007a	2.704 ± 0.004c	3.141 ± 0.005b	2.888 ± 0.003b	2.900 ± 0.004b	2.837 ± 0.003c	2.981 ± 0.003a
C17:0	0.887 ± 0.001	0.632 ± 0.001d	0.936 ± 0.001a	0.751 ± 0.001c	0.805 ± 0.001b	0.847 ± 0.001b	0.833 ± 0.002c	1.288 ± 0.001a	0.803 ± 0.001d
C17:1n7c	0.615 ± 0.001	0.457 ± 0.001d	0.713 ± 0.001a	0.531 ± 0.001c	0.584 ± 0.001b	0.547 ± 0.001b	0.469 ± 0.001d	0.603 ± 0.001a	0.531 ± 0.001c
C18:0	7.690 ± 0.014	8.540 ± 0.013b	7.125 ± 0.008d	7.842 ± 0.012c	9.373 ± 0.014a	9.499 ± 0.012b	9.381 ± 0.010c	9.245 ± 0.012d	9.727 ± 0.013a
C18:1n9c	18.798 ± 0.017	17.200 ± 0.036c	17.819 ± 0.033b	12.993 ± 0.018d	18.706 ± 0.027a	18.154 ± 0.024a	15.911 ± 0.022c	15.015 ± 0.016d	17.300 ± 0.014b
C18:2n6c	9.135 ± 0.012	17.791 ± 0.021a	8.614 ± 0.014b	7.694 ± 0.009c	6.738 ± 0.009d	7.379 ± 0.011a	7.250 ± 0.010b	5.648 ± 0.006c	3.991 ± 0.005d
C18:3n3c	1.265 ± 0.002	1.517 ± 0.002a	1.121 ± 0.001b	0.793 ± 0.001d	0.891 ± 0.001c	0.863 ± 0.002c	0.909 ± 0.001b	0.977 ± 0.001a	0.722 ± 0.001d
C20:0	0.364 ± 0.000	0.284 ± 0.000d	0.474 ± 0.001b	0.318 ± 0.000c	0.492 ± 0.001a	0.438 ± 0.001c	0.441 ± 0.001b	0.363 ± 0.001d	0.533 ± 0.001a
C20:1n9c	0.517 ± 0.000	0.363 ± 0.001d	0.525 ± 0.001a	0.446 ± 0.001c	0.458 ± 0.001b	0.441 ± 0.000b	0.495 ± 0.001a	0.395 ± 0.001d	0.428 ± 0.001c
C20:2n6c	0.592 ± 0.001	0.875 ± 0.001a	0.436 ± 0.001d	0.488 ± 0.001c	0.658 ± 0.001b	0.824 ± 0.001a	0.736 ± 0.001c	0.530 ± 0.001d	0.744 ± 0.001b
C20:4n6c	2.465 ± 0.004	2.889 ± 0.005c	2.581 ± 0.003d	3.396 ± 0.003b	3.670 ± 0.003a	3.422 ± 0.003d	3.556 ± 0.004a	3.520 ± 0.006b	3.463 ± 0.004c
C20:3n3c	0.234 ± 0.000	0.154 ± 0.000b	0.116 ± 0.000c	0.091 ± 0.000d	0.289 ± 0.000a	0.202 ± 0.000b	0.148 ± 0.000d	0.232 ± 0.001a	0.151 ± 0.000c
C22:0	0.131 ± 0.000	0.083 ± 0.000d	0.178 ± 0.000b	0.094 ± 0.000c	0.324 ± 0.001a	0.252 ± 0.000b	0.193 ± 0.000d	0.194 ± 0.000c	0.365 ± 0.000a
C20:5n3c	6.738 ± 0.006	6.672 ± 0.009d	8.055 ± 0.009c	9.708 ± 0.014a	8.499 ± 0.012b	7.972 ± 0.013d	9.312 ± 0.014c	10.721 ± 0.014a	9.404 ± 0.010b
C22:2n6c	0.317 ± 0.001	0.253 ± 0.000d	0.360 ± 0.000b	0.288 ± 0.000c	0.413 ± 0.001a	0.288 ± 0.000c	0.194 ± 0.000d	0.318 ± 0.001a	0.293 ± 0.001b
C24:0	0.236 ± 0.000	0.116 ± 0.000d	0.483 ± 0.000b	0.226 ± 0.000c	0.734 ± 0.001a	0.638 ± 0.001b	0.538 ± 0.001c	0.532 ± 0.001d	0.908 ± 0.001a
C22:6n3c	11.908 ± 0.018	10.781 ± 0.017d	17.657 ± 0.025c	24.012 ± 0.020a	18.010 ± 0.017b	19.469 ± 0.032d	21.311 ± 0.024b	20.030 ± 0.024c	23.348 ± 0.035a

Table S8. Alpha diversity indices of gut microbiota in Thamnaconus septentrionalis fed different diets

Index	K	P	X	Y
Shannon Index	5.99±0.21d	7.45±0.12b	6.94±0.25c	8.33±0.34a
Chao1 Index	577.15±16.28c	714.69±32.26b	665.03±29.00bc	794.00±36.66a
Ace Index	592.22±16.20c	740.46±25.42b	694.26±20.09bc	835.82±7.14a

Table S9. Economic cost analysis of the four experimental feeds for Thamnaconus septentrionalis culture

Parameter	K (Commercial pellet)	P (Custom formulated)	X (Frozen shrimp)	Y (Fresh fish)
Estimated feed cost (¥/ton)	8,500	12,800	15,000	18,000
Dry matter content (%)	89.5	89.2	23.8	21.5
Feed cost per ton DM (¥/ton DM)	9,497	14,350	63,025	83,721
FCR (DM basis, Day 60)	1.70	1.40	1.31	1.14
Feed cost per kg gain (¥/kg)	16.14	20.09	82.56	95.44
Weight gain (g/fish, Day 60)	33.06	52.98	46.83	56.48
Fish value at harvest (¥/kg)*	80-100	100-120	90-110	100-120
Cost-benefit ratio**	1:4.96	1:4.97	1:1.09	1:1.05

Note: Feed costs are estimated based on 2023-2024 market prices in Fujian Province, China. * Fish market value ranges reflect size- and quality-dependent pricing for T. septentrionalis. ** Cost-benefit ratio = fish sale value / total feed cost per fish over 60-day trial. DM = dry matter. Despite higher per-ton feed costs, the P group achieved the most favorable cost-benefit ratio among formulated feeds due to superior FCR and growth performance.

Table S10. Hold-out validation performance of RF-PLSR growth prediction models

Target Variable	Time Point	CV R^2 (training)	Hold-out R^2	CV RMSE	Hold-out RMSE	Hold-out MAE	Hold-out n
WGR (%)	Day 30	0.612	0.487	9.76	12.83	10.21	7
SGR (%/day)	Day 30	0.523	0.521	0.31	0.38	0.29	7
WGR (%)	Day 60	0.468	0.402	15.22	18.45	14.87	10
SGR (%/day)	Day 60	0.445	0.418	0.24	0.29	0.22	10

Note: CV = leave-one-out cross-validation on the training set (80% of samples). Hold-out = independent validation on reserved test set (20% of samples, stratified by treatment group). R^2 = coefficient of determination; RMSE = root mean squared error; MAE = mean absolute error. WGR = weight gain rate; SGR = specific growth rate. The moderate decrease in R^2 from CV to hold-out is expected given the small sample size and indicates the models capture genuine biological patterns while acknowledging limited generalizability. Bootstrap resampling (1000 iterations) was used during training to stabilize feature selection.

Figure S1. Hold-out validation of RF-PLSR growth prediction models.

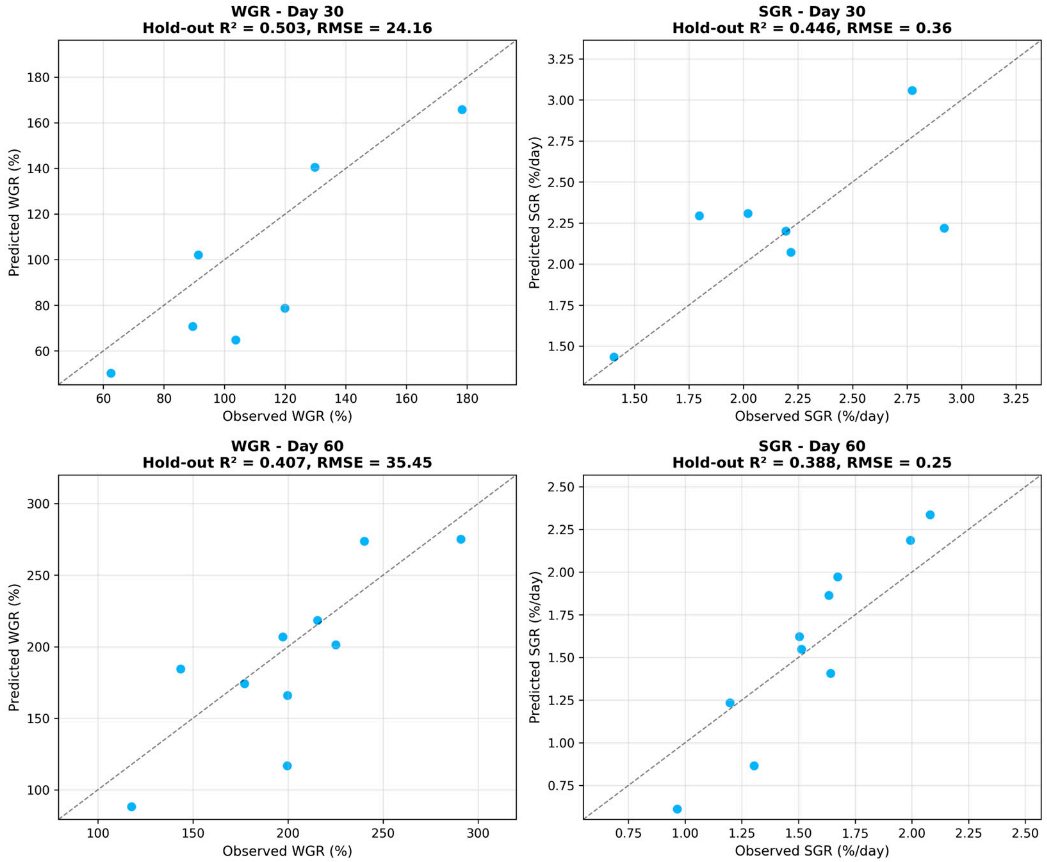

Scatter plots of observed vs. predicted values for (A) WGR at Day 30, (B) SGR at Day 30, (C) WGR at Day 60, and (D) SGR at Day 60 on the hold-out validation set (20% of total samples, reserved prior to model training). Dashed lines represent the 1:1 identity line. R^2 and RMSE values for each target variable are shown in the panel titles. The moderate decrease in R^2 from cross-validation to hold-out is expected given the small sample size and confirms that the models capture genuine biological patterns while acknowledging limited generalizability. WGR = weight gain rate; SGR = specific growth rate.
